# Genetically-defined novel oral squamous cell carcinoma cell lines for the development of molecular therapies

**DOI:** 10.18632/oncotarget.8533

**Published:** 2016-04-01

**Authors:** Muhammad Zaki Hidayatullah Fadlullah, Ivy Kim-Ni Chiang, Kalen R. Dionne, Pei San Yee, Chai Phei Gan, Kin Kit Sam, Kai Hung Tiong, Adrian Kwok Wen Ng, Daniel Martin, Kue Peng Lim, Thomas George Kallarakkal, Wan Mahadzir Wan Mustafa, Shin Hin Lau, Mannil Thomas Abraham, Rosnah Binti Zain, Zainal Ariff Abdul Rahman, Alfredo Molinolo, Vyomesh Patel, J. Silvio Gutkind, Aik Choon Tan, Sok Ching Cheong

**Affiliations:** ^1^ Cancer Research Malaysia, Subang Jaya, Selangor, Malaysia; ^2^ Oral Cancer Research and Co-ordinating Centre (OCRCC), Faculty of Dentistry, University of Malaya, Kuala Lumpur, Malaysia; ^3^ Medical Scientist Training Program, University of Colorado Denver, Aurora, CO, USA; ^4^ Oral and Pharyngeal Cancer Branch, National Institutes of Health, Bethesda, MD, USA; ^5^ Department of Oro-Maxillofacial Surgery and Medical Sciences, Faculty of Dentistry, University of Malaya, Kuala Lumpur, Malaysia; ^6^ Department of Oral and Maxillofacial Surgery, Hospital Kuala Lumpur, Kuala Lumpur, Malaysia; ^7^ Stomatology Unit, Institute for Medical Research, Kuala Lumpur, Malaysia; ^8^ Department of Oral and Maxillofacial Surgery, Tengku Ampuan Rahimah Hospital, Klang, Selangor, Malaysia; ^9^ Division of Medical Oncology, School of Medicine, University of Colorado, Aurora, CO, USA

**Keywords:** oral squamous cell carcinoma, gene expression, mutation, copy number alteration, cell lines

## Abstract

Emerging biological and translational insights from large sequencing efforts underscore the need for genetically-relevant cell lines to study the relationships between genomic alterations of tumors, and therapeutic dependencies. Here, we report a detailed characterization of a novel panel of clinically annotated oral squamous cell carcinoma (OSCC) cell lines, derived from patients with diverse ethnicity and risk habits. Molecular analysis by RNAseq and copy number alterations (CNA) identified that the cell lines harbour CNA that have been previously reported in OSCC, for example focal amplications in 3q, 7p, 8q, 11q, 20q and deletions in 3p, 5q, 8p, 18q. Similarly, our analysis identified the same cohort of frequently mutated genes previously reported in OSCC including *TP53, CDKN2A, EPHA2, FAT1, NOTCH1, CASP8* and *PIK3CA*. Notably, we identified mutations (*MLL4, USP9X, ARID2*) in cell lines derived from betel quid users that may be associated with this specific risk factor. Gene expression profiles of the ORL lines also aligned with those reported for OSCC. By focusing on those gene expression signatures that are predictive of chemotherapeutic response, we observed that the ORL lines broadly clustered into three groups (cell cycle, xenobiotic metabolism, others). The ORL lines noted to be enriched in cell cycle genes responded preferentially to the CDK1 inhibitor RO3306, by MTT cell viability assay. Overall, our in-depth characterization of clinically annotated ORL lines provides new insight into the molecular alterations synonymous with OSCC, which can facilitate in the identification of biomarkers that can be used to guide diagnosis, prognosis, and treatment of OSCC.

## INTRODUCTION

Head and neck cancer is the sixth most common cancer worldwide [1]. Collectively head and neck cancer refers to a heterogeneous group of tumors that originate from various tissue types along the upper aerodigestive tract. With a 5-year survival rate of 50–60%, oral squamous cell carcinoma (OSCC) is among the most devastating head and neck cancer subtypes [2, 3]. Unfortunately, OSCC is common especially in South-Central and South-East Asia [2] and in countries such as India, Sri Lanka, Pakistan and Bangladesh, OSCC is the most common cancer among men [1, 2].

The genomic landscape of OSCC has recently been brought to light through large scale efforts to deep sequence clinical samples. This has led to the identification of genes and pathways that could potentially be targeted with therapeutics [4–6]. To further identify actionable genes/pathways and rapidly screen potential chemotherapeutics, new experimental models are needed. Indeed, currently available *in vitro* models of OSCC may not accurately reflect the *in vivo* characteristics of OSCC, given that the majority of these models have not been subjected to detailed genetic analysis and have not been linked to detailed clinical data. The few efforts made to ensure conservation between OSCC tissues and experimental models have been limited to mutational analysis of selected genes or to a probe-based capture techniques [7, 8].

Cancer cell lines are powerful and robust experimental tools used for understanding how genetic alterations lead to tumor initiation and progression. Acknowledging the challenges associated with cost and ethics of direct clinical trials, Barrentina (2012) and Garnett (2012) utilized massive screening of chemotherapeutics in cancer cell lines to establish pharmacogenomic relationships revealing the potential biomarkers that may predict responses to drug treatment [9, 10]. Given the fact that individual genomic aberration can substantially influence therapeutic response, it is of concern that only few (~2%, 25/1292) OSCC lines were characterized in these studies. In addition, most established OSCC lines have unclear demographic details and only a few of these have been derived from patients in geographical regions where incidence rates are at its highest, such as South-East Asia [11, 12]. Furthermore, the genomic properties of many OSCC lines were not compared to the original tissues from which they were derived and many were used without full knowledge of their authenticity, which could result in false representation of the disease [13]. Hence, a complete atlas of genomic alterations in cancer cell lines is essential for their optimal use in laboratory settings, particularly in the interpretation of emerging preclinical activity of therapeutic agents that are under development.

Building on prior efforts in establishing and characterizing head and neck cancer models [13], we have established and characterized the genomic and transcriptomic alterations of a substantial number of OSCC cell lines and primary cultures from the normal oral mucosa, which we have designated as the ORL series. This panel includes cell lines from a wide range of anatomical sites of the oral cavity, those derived from patients with typical risk habits and from a growing subset of patients without any known risk habits [12, 14]. We revealed that the ORL cell lines exhibit key similarities with OSCC tissues capturing key chromosomal, mutational and gene expression aberrations. Further, the growth of ORL cell lines in subcutaneous and orthotopic-tongue xenograft mouse models demonstrated. The ORL series is set apart from previously established lines in that panel reported here have well-characterized clinical and genetic background. In line with addressing the emerging need to increase the heterogeneous representation of cell line disease models, this panel of novel OSCC cell lines will provide a valuable tool in the understanding of OSCC progression and development of molecular targeted therapies in the era of precision oncology.

## RESULTS

### Establishment of oral cancer cell lines

**Figure 1:**
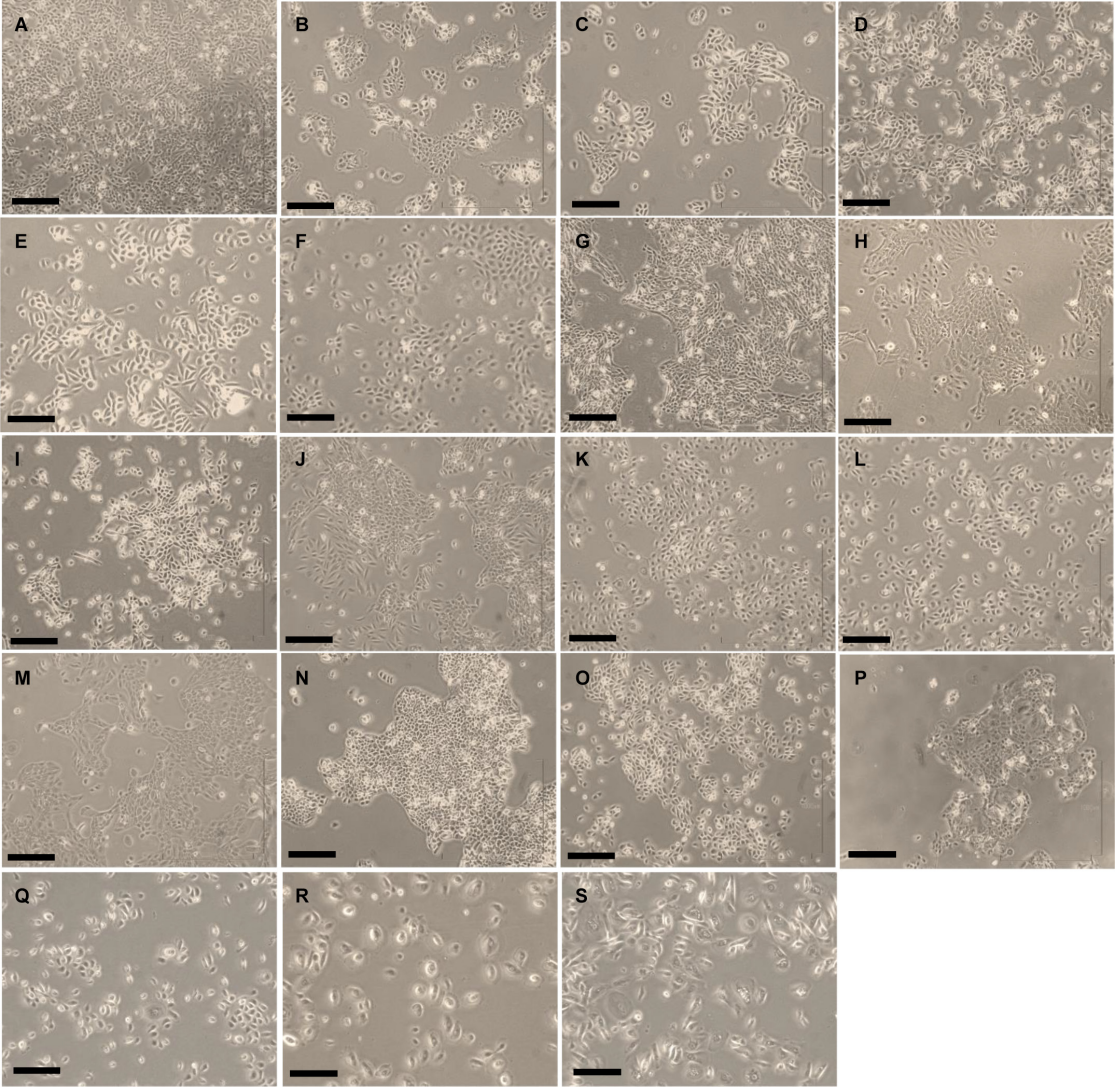
Morphological appearance of the ORL lines Phase contrast micrograph of (**A**) ORL-48, (**B**) ORL-115, (**C**) ORL-136, (**D**) ORL-150, (**E**) ORL-153, (**F**) ORL-156, (**G**) ORL-166, (**H**) ORL-174, (**I**) ORL-188, (**J**) ORL-195, (**K**) ORL-196, (**L**) ORL-204, (**M**) ORL-207, (**N**) ORL-214, (**O**) ORL-215, (**P**) ORL-247, (**Q**) ORL-232N, (**R**) ORL-235N, (**S**) ORL-231N. Bar = 500 μm.

**Figure 2:**
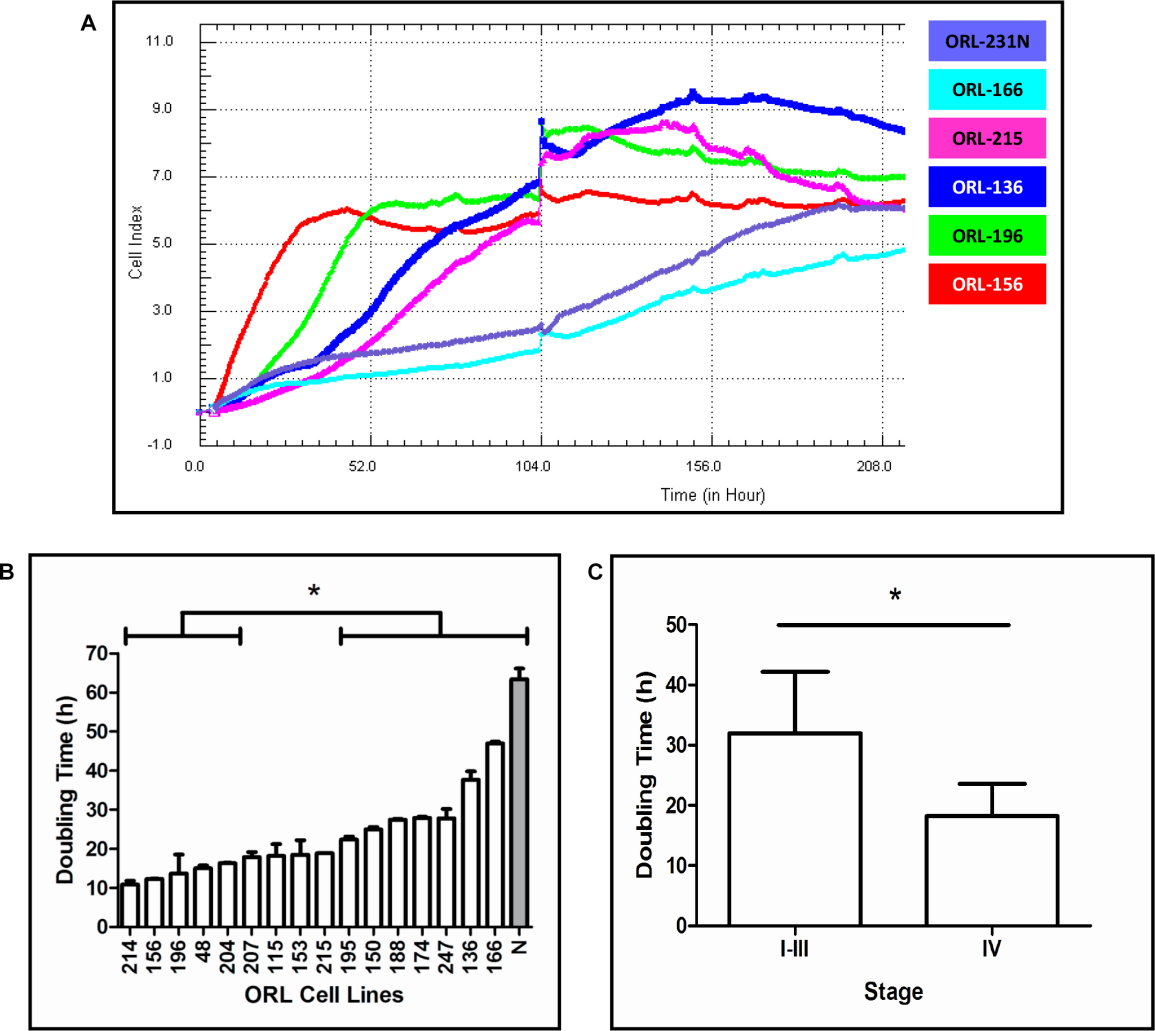
Growth properties of ORL lines (**A**) Growth curves of ORL cell lines. Cell lines with short (ORL-156, ORL-196), intermediate (ORL-136, ORL-215) and extended (ORL-166, normal keratinocytes ORL-231N) lag phases representing the ORL series are shown. (**B**) Proliferation rates of ORL lines. Cell lines with fast, intermediate, and slow proliferation rates are shown from left to right. All bars contained within a bracket set are significantly different than bars contained within the comparison bracket set. Normal oral keratinocytes from 3 different primary cultures are shown in the grey bar. Population doubling was calculated from an average of 2–3 experiments. (**C**) Mean doubling times (h) of cell lines derived from stage I-III and stage IV tumors. *denotes significance of *p* < 0.05.

In an effort to establish OSCC cell lines that are molecularly representative of clinical specimens, OSCC tumor and normal gingival tissues were processed as previously described to establish primary cultures [15]. From this initiative, we successfully established 16 spontaneously immortalized cell lines derived from the OSCC tumor tissues (referred to as ORL cell lines) that have undergone more than 100 population doublings, while the normal oral keratinocyte (NOK) cultures senesced when they were maintained beyond passage five or six. Our first analysis with the ORL cell lines was to perform STR profiles to ascertain base line authenticity and this was demonstrated, with the data giving a match of > 85% to the respective donors (Supplementary Table S1). This series of cell lines were derived from the most common anatomical sites of the oral cavity and were from patients with diverse etiological factors including tobacco smoking, betel quid chewing (smokeless tobacco) and alcohol consumption (Table 1). Microscopic evaluation of cell lines show that they are polygonal in shape, grow in monolayers and exhibit cobblestone-like morphology typical of keratinocytes (Figure 1). Of all the cell lines, only one (ORL-115) was tested positive for HPV (types 16 and 31; data not shown). Consistent with the immortal properties associated with cancer cell lines, ORL cell lines showed high telomerase activity levels as determined by TRAPeze assay relative to NOK primary cultures (Supplementary Table S2). Growth of these cells was monitored in real-time to document the growth rates of the ORL cell lines for future utilization of these cells. Representative growth curves are shown in Figure 2A and broadly, ORL cell lines have doubling times between 10.9–47.0 hours (Figure 2B). Notably, those cell lines derived from stage IV tumors demonstrated significantly shorter average doubling times (18.2 hours) compared to lines derived from stage I to III tumors (32.0 hours; *p* = 0.036; Figure 2C).

**Table 1:** Demographic details of the patients from whom the ORL lines were derived

Line Designation	Age at diagnosis	Gender	Ethnicity	Oral habits^a^	Primary tumor site^b^	TNM (Stage)^c^	Patient status
ORL-48	79	F	Indian	None	G	4, 2, 0 (IV)	Deceased
ORL-115	75	F	Indian	BQ	G	4, ×, 0 (IV)	Deceased
ORL-136	56	M	Indian	BQ, T, A	T	1, 0, × (I)	Unknown
ORL-150	76	M	Indian	A	T	1, 0, × (I)	Recurrent disease
ORL-153	36	M	Indian	T	G	4, 2, × (IV)	Deceased
ORL-156	38	M	Chinese	T, A	T	1, 2, 0 (IV)	Deceased
ORL-166	66	F	Malay	None	T	2, 1, 0 (III)	Deceased
ORL-174	53	F	Indian	BQ	T	2, 0, 0 (II)	Free of disease^e^
ORL-188	56	M	Malay	T	T	2, 2, × (IV)	Deceased
ORL-195	61	F	Indian	BQ	BM	2, 0, × (II)	Deceased
ORL-196	59	F	Indian	BQ, A	BM	2, 2, × (IV)	Free of disease^e^
ORL-204	76	M	Indian	BQ, T, A	BM	4, 1, × (IV)^d^	Deceased
ORL-207	63	F	Indian	BQ	T	1, 2, 0 (IV)	Deceased
ORL-214	49	F	Indian	BQ	BM	4, 0, × (IV)	Free of disease^e^
ORL-215	50	M	Indian	T	T	4, 2, × (IV)	Deceased
ORL-247	38	M	Indian	T, A	T	4, 2, × (IV)	Deceased

a BQ = betel quid chewing; T = tobacco smoking; A = alcohol drinking.

b G = Gingiva; BM = buccal mucosa; T = tongue.

c Largest tumor dimension and and node status determined by histopathology examination.

d Tumor size and node status determined by clinical examination-patient was treated with radiotherapy.

e Disease free after.

5 year follow-up.

### Tumorigenicity of ORL lines in animal models

To determine the utility of these cell lines in *in vivo* models, a sub-set of these were chosen (ORL-48, -115, -136, -150, -174, -188 and -204) to evaluate their subcutaneous and orthotopic growth in the flank and tongue, respectively. The cell lines were chosen to represent those derived from patients with distinct risk habits, subsites, and those representing different gene expression clusters (see below). In the subcutaneous flank xenograft study, two lines from this sub-set (ORL-48, ORL-115) formed solid and palpable tumors. The tumor take rate for ORL-48 and -115 were 73.5% and 65%, respectively (Table 2A). Notably, histopathological evaluation of these xenograft tumors, show features that essentially resemble those of the primary tumors from patients (Supplementary Figure S1A). Growth kinetics of these two cell lines were shown in Supplementary Figure S1B. ORL-48 has a tumor volume doubling time of 6 days and achieved a tumor volume of 150 mm^3^ at 36 days post xenograft. Meanwhile, ORL-115 tumor has a relatively longer lag phase with a tumor volume doubling time of 10 days, and only reached 150 mm^3^ at 70 days post-xenograft. Tumor burden for the remaining five ORL cell lines analyzed (ORL-136, ORL-150, ORL-174, ORL-188, ORL-204) was minimal after 60 days (< 40 mm^3^) and with some observed to regressed, likely indicating non-tumorigenic nature of the cells in the subcutaneous xenograft model.

**Table:** Growth characteristics of ORL lines in subcutaneous xenograft model

Cell lines	Subcutaneous take rate (%)	Time to reach 150 mm^3^ (Days)
ORL-48	25/34 (73.5)	36
ORL-115	13/20 (65)	70
ORL-136	2/34 (5.9)	NA
ORL-150	0/42 (0)	NA
ORL-174	0/30 (0)	NA
ORL-188	0/34 (0)	NA
ORL-204	0/36 (0)	NA

NA: Data not available as these tumors did not exceed the tumor volume of 40 mm^3^.

The same cohort of cell lines used for subcutaneous model, were also examined in the orthotopic model to recapitulate the environment of OSCC. Six of the seven ORL cell lines (ORL-48, ORL-115, ORL-136, ORL-150, ORL-188, ORL-204) formed solid and palpable tumors in the tongue of the animals with tumor take of 70–100% (Table 2B and Supplementary Figure S1C). The growth rate of these lesions were broadly constant over time and exceeded tumor volumes of 10 mm^3^ within 15 to 33 days (Table 2B). Although ORL-174 did not form palpable tumors in the orthotopic tongue xenograft model, histopathological evaluation on the tongues of these animals indicated that the underlying connective tissues in the tongues were infiltrated with moderately differentiated tumor cells in a focal area (Supplementary Figure S1C). Since human OSCC typically metastasizes to the cervical lymph nodes, we examined the cervical lymph nodes of the mice bearing tongue lesions. We showed that ORL-48 and ORL-150 cells were readily metastatic, as indicated by the presence of tumor cells in cervical lymph nodes of the animals injected with these cells (Supplementary Figure S1D). Lymph node metastasis was not observed for all the other ORL cell lines that were tested.

**Table:** Growth characteristics of ORL lines in orthotopic tongue xenograft model

Cell lines	Orthotopic take rate (%)	Time to develop palpable tumor 10 mm^3^ (Days)	Tumor histopathological features	Lymph nodes histopathological features
ORL-48	15/15 (100)	15	Moderately differentiated tumors showing perineural, intramuscular and intravascular infiltration	OSCC metastasis
ORL-115	12/17 (70.6)	33	Well differentiated tumors showing intramuscular infiltration	Hyperplastic
ORL-136	15/16 (93.8)	28	Moderately differentiated tumors showing perineural, and intramuscular infiltration	Hyperplastic
ORL-150	12/12 (100)	23	Moderately differentiated tumors	OSCC metastasis
ORL-174	0/11 (0)	NA	Moderately differentiated tumors at a focal area showing intramuscular infiltration	Hyperplastic
ORL-188	11/11 (100)	19	Moderately differentiated tumors showing perineural and intramuscular infiltration	Hyperplastic
ORL-204	11/11 (100)	23	Moderately differentiated tumors showing intramuscular infiltration	Hyperplastic

NA: Data not available as no formation of tumor is observed.

### Global molecular profiles of ORL lines closely resemble OSCC specimens

**Figure 3:**
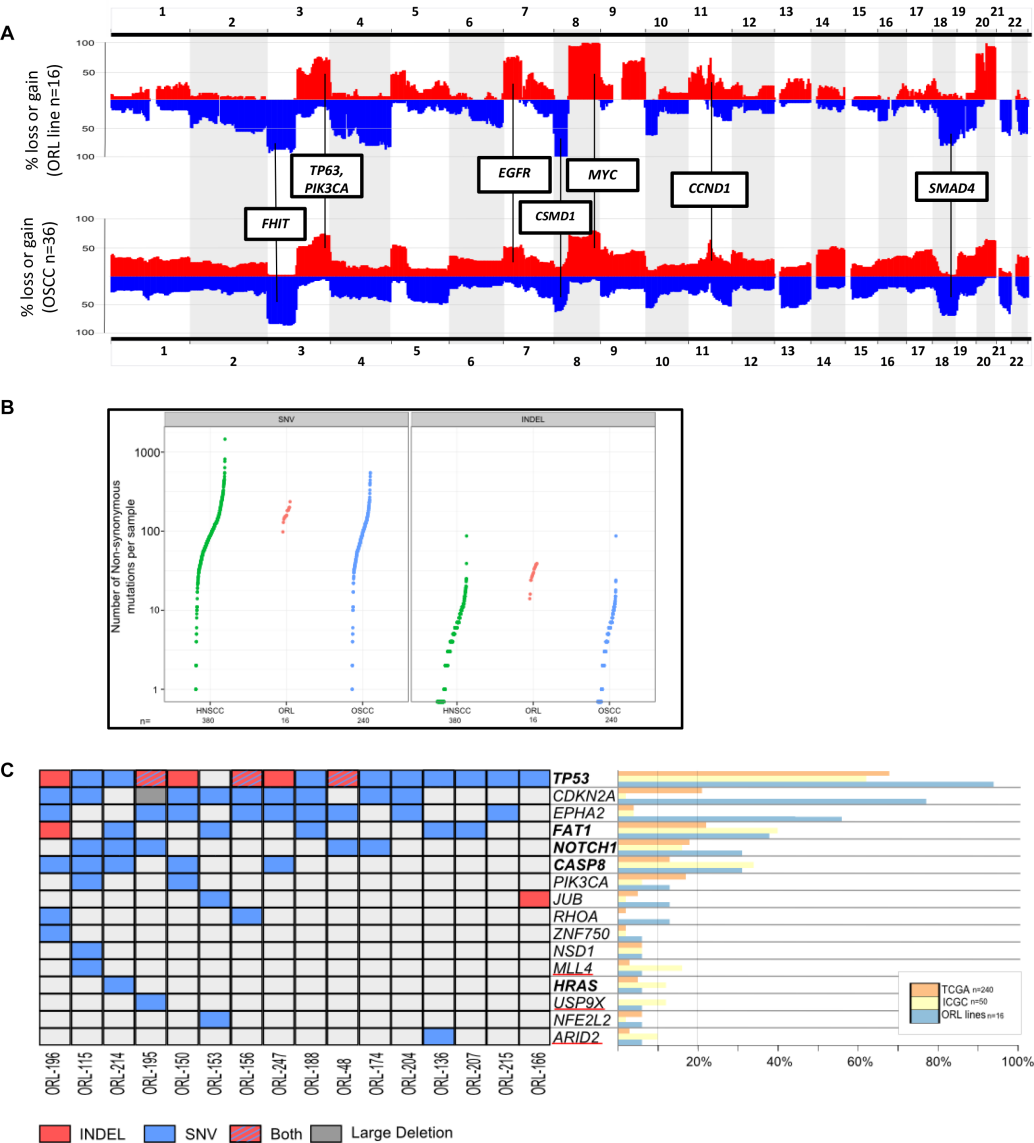
Global profiling of copy number alterations (CNA) and mutations in ORL lines (**A**) Genome-wide frequency distribution of CNA in 16 ORL tumor lines (top) and 36 oral squamous cell carcinoma patients obtained from Pickering et al. 2013 (PMID:23619168, bottom). Amplification are shown in red and deletions are shown in blue. (**B**) Mutation frequency in ORL lines (Red) compared to HNSCC (Green) and OSCC tissues (Blue). Mutation frequency in tissues were obtained from TCGA (PMID: 24390350). (**C**) Representation of mutations detected in the ORL lines in top mutated genes previously reported in HNSCC. Genes listed are obtained from TCGA and ICGC study. Common significant genes found in both data sets are indicated in bold and genes only mutated in ICGC study are underlined in red. Bar graph to the right tabulates the frequency of the genes found to be mutated in oral cancer samples. A comprehensive list of mutations in the ORL cell lines are tabulated in Supplementary Table S4. TCGA - The Cancer Genome Atlas (PMID: 24390350); ICGC - International Cancer Genome Consortium (PMID: 24292195).

To date, OSCC cell lines have been primarily characterized based on their cytokeratin expression, candidate genes expression and more recently by microarray and next generation sequencing based platforms [7, 9, 10]. As part of our effort to catalogue the global molecular profile of these novel OSCC cell lines in an unbiased way, ORL cell lines together with NOK primary cultures were comprehensively analyzed by copy number profiling and RNAseq. Chromosomal instability in the form of copy number alterations is a common feature of human cancers, and OSCC are amongst those reported to be particularly driven by copy number alterations [16]. We noted from our analysis that the ORL cell line panel have major chromosomal abnormalities, with a mean of 17.5 copy number alteration (CNA) per cell line. Thirteen of the lines (81%) have at least 16 CNAs, indicating high genomic instability as previously reported for OSCC [17]. Of note, ORL-115, the only HPV positive line has among the least focal and arm level CNAs (*n* = 12) compared to other HPV negative lines (range 12–24). Based on prior studies looking at CNA in head and neck cancers [5, 18], we demonstrated that the CNAs identified in the ORL cell line panel were highly similar to those commonly found in head and neck squamous cell carcinoma (HNSCC) tissues (Figure 3A), including focal gains (3q, 7p, 8q, 9q, 11q, 20q) and losses (3p, 5q, 8p, 18q; Supplementary Table S3). This demonstrates that key genes involved in OSCC development are likely recapitulated in the ORL cell lines. These regions of CNA alterations contain candidate and established oncogenes as well as tumor suppressors. In this context, we observed the gains of *TP63* (62.5%),*PIK3CA* at 3q (62.5%), *EGFR* at 7p (69%), *MYC* at 8q (100%) and *CCND1* at 11q13 (62.5%). Losses on the other hand included *FHIT* at 3p (56.3%), *CSMD1* at 8p (81.3%) and *SMAD4* at 18q (56.3%; Figure 3A). However, unlike most OSCC, disruption by focal deletion in the 9p chromosomal region where the tumor suppressor *CDKN2A* is located was not observed in any of ORL cell lines. Instead *CDKN2A* was noted to be frequently disrupted by somatic mutations in these lines as discussed below. All of the genes that fall within the described CNA regions are listed in Supplementary Table S3.

Mutational status is a significant factor that can modulate therapeutic responses, therefore we compared the mutational profiles from the ORL cell line panel to 380 HNSCC tumors tissues (240 of these constituting OSCC) recently reported by The Cancer Genome Atlas (TCGA) [19]. We found that the number of SNV (98–236) and INDELs (14–39) in the ORL cell lines, were similar to the range of mutations reported in the TCGA (Figure 3B), demonstrating the conservation of overall mutation rates. It is noteworthy to mention that although our analysis lack matching normal DNA to make valid call for somatic mutations, the frequency of mutations in the ORL cell lines are comparable to those reported for OSCC tissues specimens. We did not observe differences in the distribution of transversion and transition mutations in relation to the various risk habits of the patients from which the cell lines were derived (Supplementary Figure S2A). All mutated genes for the ORL cell lines are catalogued in Supplementary Table S4 for referencing when utilizing these cell lines.

Based on the significantly mutated genes reported for HNSCC specimens in both the TCGA and the International Cancer Genome Consortium (ICGC) [20], we found these were largely represented in the RNAseq data of the ORL cell lines (Figure 3C). *TP53* and *CDKN2A* were amongst the most frequently mutated genes in the ORL cell lines (94% and 63% respectively). As the mutation status of these genes can add value by improving our overall understanding of tumor biology and influence on drug response [21, 22], we validated a sub-set of these mutated genes by Sanger sequencing. To this end, we sequenced exons 4–11 of the *TP53* and exons 1–3 of the *CDKN2A* genes and essentially validated the mutations identified from RNAseq. Notably, the only HPV positive line (ORL-115) also harbored a *TP53* mutation. To determine the somatic origin of these mutations and to provide an addition level of authentication, mutations in *TP53* and *CDKN2A* in ORL lines were compared to those from the original tissue specimens where available. Of the mutations in fresh frozen tissues examined, 92% (11/12) have *TP53* status identical to their corresponding cell lines (Supplementary Table S5). Further, of the *CDKN2A* mutations in ORL lines, we found 77% (10/13) harbored *CDKN2A* genotypes that were identical to the patient-matched sample (Supplementary Table S6). This suggests that some *CDKN2A* mutations in three of the ORL cell lines are likely culture induced. We were unable to conduct concordance analysis in all samples due to limited genetic material. As all ORL cell lines are essentially immortal, this is in line with previous reports that inactivation of *TP53* and *CDKN2A* are important events in overcoming cellular senescence [23].

While the significant mutations identified in both the TCGA and ICGC studies are well-represented in the ORL cell line panel, it was of interest to note that the frequency of specific mutations, for example *FAT1* and *CASP8* were higher in both the ORL and ICGC samples compared to those observed in the TCGA study alone (Figure 3C). This may be explained in part by patient demographics for instance, ORL samples have similar profiles as the ICGC samples that of Indian origin, likely with betel quid chewing habits, and focused specifically on gingiva-buccal tumors, while the TCGA cohort is mainly of Caucasian origin where betel quid is not the major etiological factor and majority of the specimens were from the tongue and floor of the mouth. Further, genes that were found to be significantly mutated in the ICGC study (*MLL4*, *USP9X, ARID2*), were represented only in ORL cell lines that were derived from patients with a betel quid chewing habit and not in those with a history of smoking, suggesting that the genes may be reflective of distinct etiological factors.

**Figure 4:**
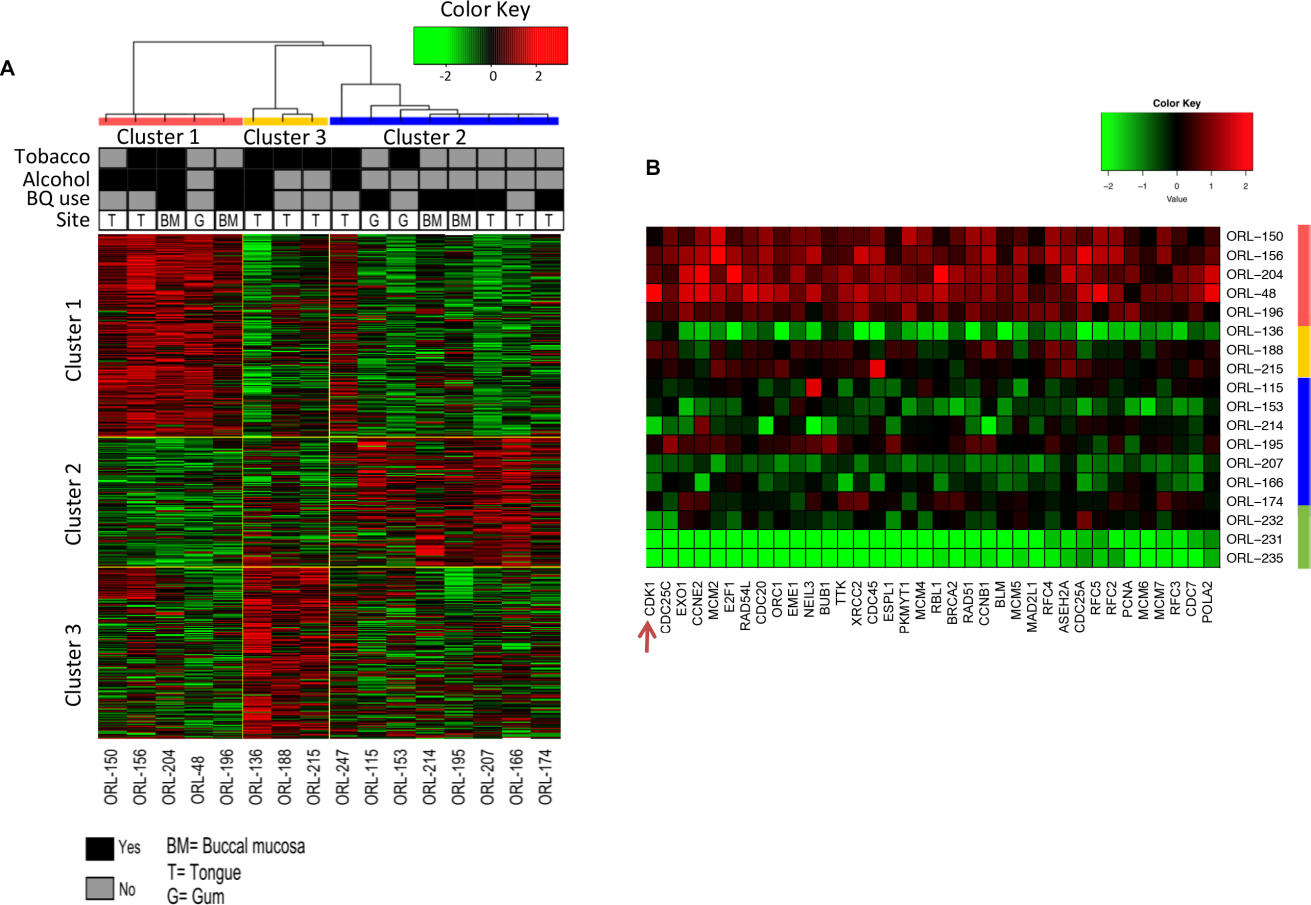
Genomic profiling reveals three distinct subgroups in ORL lines (**A**) Unsupervised clustering by Consensus Cluster revealed the presence of three subgroups denoted by the three colors (Red, Yellow and Blue). Risk habits and tissue site of origin are shown. Heatmap are of genes representative in each subgroup's pathway as determined by Gene Set Enrichment Analysis. A comprehensive list of pathway and gene list is detailed in Supplementary Table S5. (**B**) A total of 36 genes were found to be enriched (> 5 fold) in the top ten pathways in Cluster 1 compared to normal oral keratinocytes. Genes are arranged from the most up-regulated from left to right. CDK1 (shown by arrow) appears to be the most highly expressed gene in Cluster 1, relative to normal oral keratinocytes. Site: T = Tongue; BM = Buccal mucosa; G = Gum.

Our RNAseq analysis revealed a distinct expression profile of the ORL cell line panel and the NOK primary cultures (Supplementary Figure S2B). Further analysis then focused on the identification of major molecular pathways in the ORL cell lines and whether they recapitulated expression patterns previously reported for OSCC. To this end, observation from consensus clustering [24] and Principal Component Analysis (Supplementary Methods) broadly divided the ORL cell lines into three distinct clusters (*k* = 3; Supplementary Figure S3A–S3E). To further determine the molecular basis of each subgroup, we performed Gene Set Enrichment Analysis (GSEA) [25] and revealed enriched pathways within each of the three subgroups (Figure 4A) and these have been previously reported to define OSCC [5] and head and neck cancers [26, 27]. In line with previous reports for OSCC, Cluster 1 demonstrated significant enrichment in the regulators of the cell cycle including *CCND1* expression and upregulation of cyclin dependent kinases (CDKs; Figure 4B; Supplementary Table S7) [5, 28]. Distinct from Cluster 1, the second cluster featured pathways regulating xenobiotic metabolism. Genes such as *PPAR*, *AKR1C1* and *GSTM3* were enriched in this sub-group (Supplementary Table S7). High expression of *AKR1C1* has been previously documented as a feature in HNSCC tumors [26, 27]. Our analysis broadly illustrates the robustness of the ORL cell line panel in recapitulating previously reported enriched sub-groups in HNSCC tumors. Finally, three other ORL cell lines were grouped in Cluster 3. We were not able to robustly identify enriched pathways within this group likely due to the inherent heterogeneity in the gene expression within this small sub-set of cell lines. Notably however, extremely high EGFR expression was observed in ORL-136 (> 2 million reads mapped to EGFR) as confirmed by western blot analysis (Supplementary Figure S4). Taken together, the genetic profile of the ORL cell lines are diverse and represents the critical pathways that are well-reported in head and neck cancers forming the basis that these cell lines can be used as important models in facilitating pre-clinical evaluation of new therapeutic agents against specific genetic alterations.

### Gene expression clustering of ORL cell lines influence response to clinically relevant therapeutics

**Figure 5:**
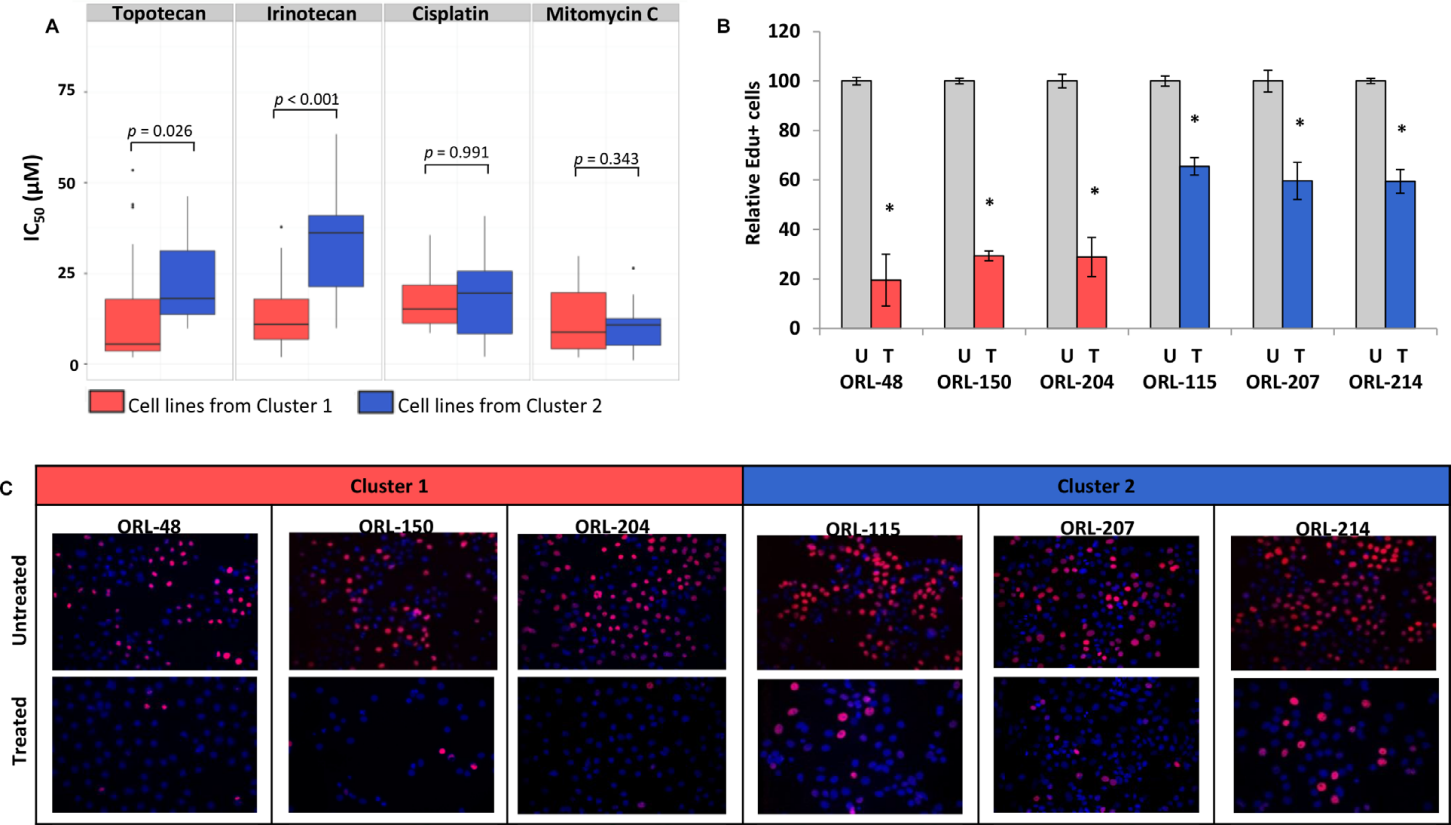
Cell lines from different gene expression clusters demonstrate differential response to specific inhibitors (**A**) Cell lines from Cluster 1 are significantly more sensitive to Topoisomerase 1 inhibitors compared to cell lines from Cluster 2 but selectivity was not observed in response towards general DNA-crosslinking agents such as cisplatin and mitomycin C. (**B**) Graph demonstrating that the inhibition of cell proliferation in cell lines from Cluster 1 is at least twice of that in cell lines from Cluster 2. (**C**) Cell lines in Cluster 1 are more sensitive to CDK1 inhibitor as demonstrated by reduced proliferation levels after treatment with CDK1 inhibitor (RO-3306) at 7 μM for 24 hours. Blue (DAPI) represents the total number of cells in any field and red represents proliferating cells that have incorporated the EdU label. *denotes significance of *p* < 0.05 as determined by Student's *t*-test.

To determine whether there is a clinical significance for the segregation of cell lines based on their gene expression clustering, we treated the ORL cell lines with a panel of clinically relevant chemo-therapeutic agents (Figure 5A; Supplementary Figure S5A). We demonstrated that overall, cell lines in Cluster 1 (enriched with cell cycle and DNA repair pathways; Supplementary Table S7) responded remarkably well to DNA Topoisomerase 1 (TOP1) inhibitors compared to cell lines from Cluster 2 (enriched for xenobiotic metabolism; Supplementary Table S7), where IC_50_ values were significantly different between the two clusters (*p* = 0.02 for irinotecan; *p* = 5.14 × 10^−8^ for topotecan; Figure 5A). Not surprisingly, we did not observe any differences in response to general DNA-crosslinking agents such as cisplatin and mitomycin C that are not selective based on distinct genetic profiles.

Based on consensus clustering and the analysis of top ten enriched pathways in Cluster 1, we found 36 genes that were significantly elevated by more than five-fold (*p* < 0.05) compared to NOKs (Figure 2B). We wanted to determine whether we could identify targetable genes within this cluster that could respond selectively to specific targeted drugs. Notably, among these genes, cyclin-dependent kinase 1 (CDK1), an essential protein in cell cycle progression was found to be the most highly differentially expressed gene in this cluster compared to cell lines from other clusters. Given that CDK1 was significantly over-expressed in OSCC tissues [28] and a sub-set of ORL cell lines, we hypothesized that ORL cell lines in Cluster 1 would be more sensitive to a CDK1 inhibitor. To test this hypothesis, three ORL cell lines respectively from Cluster 1 (ORL-48, ORL-150, ORL-204) and Cluster 2 (ORL-115, ORL-207, ORL-214) were treated with a specific CDK1 inhibitor, RO-3306, for 24 hours and inhibition of cell proliferation was measured. Inhibition of cell proliferation was more than 70% for cell lines in Cluster 1 (ORL-48, ORL-150, ORL-204) whereas for Cluster 2 (ORL-115, ORL-207, ORL-214), growth inhibition was observed in less than 40% of the cells (Figure 5B and 5C; Supplementary Figure S5B). This result supports our hypothesis that ORL cell lines in Cluster 1 which express high CDK1 levels are more sensitive to CDK1 inhibitor as compared to ORL cell lines from other clusters and these lines could be potentially used to identify therapeutic agents based on specific gene enrichment reported to be present in OSCC tissues.

## DISCUSSION

*In vitro* cancer models are important tools for understanding the functional roles of genetic drivers in cancer pathogenesis. Furthermore, these models can have clinical utility in the identification and testing of novel therapeutics particularly in the current era of personalized medicine where therapeutic response is correlated to the genetic background of the tumor. However, clinical studies have shown that high levels of heterogeneity exist even within clinically defined cancer subtypes [29, 30]. This is recently mirrored in high-throughput screening of a plethora of anti-cancer agents across hundreds of cancer cell lines where heterogeneous response to therapeutic agents varied according to genetic profiles within the same tumor type underscoring the need to have wider representation of head and neck cancer biology in the cell lines that are used to study this disease [9, 10].

Here we describe the establishment and characterization of a large panel of cell lines derived from lesions from representative sites of the oral cavity from cancer patients with exposure to diverse etiological agents including those with a history of smoking tobacco and chewing betel quid (smokeless tobacco). Although a large proportion of global oral cancer cases are associated with betel quid chewing, *in vitro* models representing these cancers are scarce and those available have yet to be fully characterized [12, 14]. Previous studies have demonstrated that the genetic progression of oral cancer differ with distinct etiological factors [31, 32]. Taking into consideration that gene expression and mutational profiles have been demonstrated to play a critical role in response to therapeutic agents, we characterized the ORL cell line panel in depth primarily to enable their use as *in vitro* models that would widen the representation of current head and neck cancer cell lines, in line with the global call to generate a cancer dependency map based on cell line models [33]. First and foremost, we provide STR profiles of newly established lines that are mandatory requirements for use as authenticated *in vitro* models, ensuring that any emerging data is robust and crucially, to prevent false representation of cell lines including those derived from head and neck cancer patients that has blighted cancer research [13].

Next, the ability to grow and form solid tumors in immunodeficient mouse is another important requirement, for cell line models to create reliable preclinical animal models for investigating OSCC pathogenesis and testing anticancer therapies. To this end, two of the seven ORL cell lines that were tested, formed solid subcutaneous tumors while six of the seven, formed orthotopic tongue tumors. Although subcutaneous tumor models offer simplified tumor establishment and tumor monitoring [34], subcutaneous growth from OSCC cell lines have been reported to be exceedingly difficult [35]. Tumor take rates were markedly improved when ORL cells were implanted orthotopically in their tissue of origin as previously described [36]. Notably, the tumors formed by the ORL cell lines faithfully reproduced tumors with local invasion and metastasis that resemble the original tumor tissues. Indeed, from our data we observed that ORL-48 and ORL-150 cells reproducibly developed primary lesions followed by local invasion and lymph node metastasis, essentially demonstrating that disease dissemination of human OSCC is recapitulated in our orthotopic xenograft model, making these useful models in investigating the genetic basis of OSCC progression.

While distilling the complex genomic alterations of multiple tumor types, recent analyses have subdivided these broadly into 2 categories, those that are primarily inundated with somatic mutations (M class) and those characterized by multiple recurrent chromosomal gains and losses (C class) [16]. On this note, majority of head and neck cancers fall within the C class of tumors and consistent with this, we observed gains on chromosomal regions 3q, 5p, 7p, 8q, 11q and 20q and losses on 3p, 8p and 18q in the ORL cell line panel, broadly recapitulating those reported for OSCC and head and neck cancers [37–39]. These chromosomal regions harbor known tumor suppressors, for example the fragile histidine triad gene (FHIT) whose loss has been demonstrated to be an early event in OSCC and closely linked with genomic instability and is a predictor of poor outcome in OSCC [40]. Further, 18q12.3-q23 region harboring *SMAD4* is lost in more than 50% of the ORL cell line panel (9/16). SMAD4 is now known to play a crucial role in the TGF-b signaling pathway and its inactivation is associated with poor-survival in OSCC patients [41]. The loss of *SMAD4* is in line with aberrations that are observed within TGF-b signaling and decreased protein levels could explain the inactivation of the TGF-b tumor suppressive pathway in HNSCC [7]. Consistent with current understanding of key oncogenic changes in OSCC, gains in the regions harboring individual key oncogenic genes including *PIK3CA* (3q25.2-q29), *EGFR* (7p21.3-p11.2) and *MYC* (8q23.3-q24.3) were also observed in majority of the ORL cell line panel. These observations underscore the fact that the most common key genetic drivers observed in OSCC and HNSCC [5, 42] are well-represented in this panel, providing new avenues for the study and understanding of genetic compromises that drive the development of OSCC.

From our RNAseq analysis, we identified a significant number of mutations present in the ORL cell line panel and notably, nine of the 10 most significant genes identified in the TCGA [19], were also represented, albeit with some variation in frequencies. Furthermore, *TP53* and *CDKN2A* were the 2 of the most common mutated genes identified in the ORL cell lines, for example aberrant *TP53* was present in 15/16 ORL lines. Co-occurrence of mutations within these 2 genes that has been reported as a unique feature in head and neck cancers [43], was faithfully confirmed in the ORL cell lines. Notably, mutations in the ORL cell lines recapitulate similar mutations in tumor tissues where mutations that were reported in the effector domain of *RHOA* were also detected in two ORL cell lines (ORL-156, ORL-196), suggesting that defects within this particular domain could regulate RHOA activity and provide a survival advantage to tumor growth [19]. Mutations in *PIK3CA* are the most common oncogenic mutations reported in OSCC and consistently, 2/16 ORL cell lines (ORL-150, ORL-115) were noted to harbor these mutations. In addition, mutations in the inhibitory subunit of the PI3K (PIK3R1) were found in an additional 3 independent cell lines (ORL-135, ORL-215, ORL-204) making PI3K the most mutated oncogenic pathway found in the ORL cell line panel.

Interestingly, we found several differences between mutation frequencies in some of the genes in the ORL cell line panel when compared to the TCGA study. For example, mutation frequencies of *FAT1* and *CASP8* were much higher in the ORL cell lines [19] and this difference is also seen when comparing the data from TCGA and ICGC, where a difference of two fold was noted for these two genes in the ICGC specimens [44]. Furthermore, several genes reported to be significantly mutated in ICGC that included *MLL4*, *USP9X* and *ARID2* were also found mutated in the ORL cell line panel, particularly those established from patients who chewed betel quid. These genes reported to be mutated in other cancers, primarily play a role in epigenetic regulation of key genes for example, *p53* and *SMAD4*. Therefore it is likely that these genes have tumor suppressive activities and loss of function could contribute to the development of OSCC [45–48]. Taken together, mutations in well-investigated genes as well as putative genetic drivers are represented in the ORL cell line panel and while several of these are now known to be consistently altered in OSCC regardless of etiological factors, several may be associated with different risk habits as reported previously [31].

Previous gene expression studies have demonstrated that HNSCC could be divided into several subtypes that can be defined by the enrichment of distinct molecular pathways. To determine whether these enriched pathways feature in the ORL cell line panel, we analyzed the RNAseq data and determined that the gene expression pattern broadly separated into 3 distinct clusters. While the identification of defined subtypes that were previously reported [49] was not statistically possible with the limited number of cell lines in this study, nevertheless, pathway enrichments previously reported for HNSCC and OSCC, were readily identified within these respective clusters. Cluster 1 enriched for cell cycle related genes including the MCM family of proteins, while Cluster 2 featured xenobiotic enzymes including those in the alcohol dehydrogenase (ALDH) and glutathione S transferase, both have been previously reported for HNSCC and OSCC [26, 27, 42, 49]. We also note that ORL-136 falling into Cluster 3 had particularly high levels of EGFR transcript, which we consequently confirmed to be an activated signaling pathway (Supplementary Figure S4). Our data suggest that the ORL cell line panel may hold value for evaluating novel therapies for OSCC, particularly those targeting mitogenic pathways including those driven by EGFR.

To determine whether the gene expression pattern segregating the ORL lines into distinct clusters have clinical significance, we treated a subset of lines with several chemotherapeutic drugs. Irinotecan and topotecan are used in the treatment of colorectal and ovarian cancers respectively [50, 51] and they cause cytotoxicity by generating DNA double strand breaks [52, 53]. One possible reason for the selective sensitivity that is observed in cell lines within Cluster 1 is that these cell lines have enriched expression of cell cycle genes including those regulating DNA replication. Cells that are constantly undergoing cell division are particularly vulnerable to topoisomerase inhibitors [54, 55] essentially due to high DNA replication activity and thus explaining in part, the selectivity demonstrated in ORL cell lines within Cluster 1. Sensitivity to topoisomerase inhibitors can also be influenced by ATP-binding cassette (ABC) transporters, defective DNA repair and/or the apoptotic machinery, however, mutations in genes regulating these pathways do not fully explain the response seen with topoisomerase inhibitors [53, 56]. To this end, cell line systems such as the ORL panel described in this study, will not only provide rationale for the testing this class of drugs in oral cancer, but could also provide a platform for delineating molecular markers that may have values to determine which tumors are likely to respond.

Of interest, we noted CDK1 to be highly expressed in the cell lines within Cluster 1 and demonstrated that these were selectively more sensitive to the CDK1 inhibitor, RO-3306. CDK1 represents a core component of the cell cycle and forms complexes with cyclin A and cyclin B to promote cell cycle progression from S to G2/M phase [57, 58] and unlike normal cells, cancer cells are highly dependent on G2/M checkpoint for genomic damage repair broadly due to a defective p53-dependent G1/S checkpoint that is inherent for cancer development [59]. The intricate involvement of CDK1 in the cell cycle and DNA repair mechanisms suggests that CDK1 could be an important target in cancer treatment [60] and more importantly, a previous study demonstrated that the effect of CDK1 inhibitor was selective towards cancer cells [61]. Taken together the data suggest that targeting CDK1 could be a viable approach in controlling OSCC and further studies to evaluate this area are warranted.

Recent studies have elucidated the genomic landscape of OSCC and provided a unique opportunity to identify many putative drivers of OSCC that may be targetable for therapeutic purposes. However, converting genetic drivers that have utility as therapeutic targets or biomarkers into the clinical setting would require experimental demonstration of oncogenic activities and a good understanding of molecular mechanisms of action. In this regard, our analysis of a panel of novel OSCC cell lines underscores some of the important genetic observations in OSCC development. More importantly, we demonstrate that the ORL cell lines could add to the existing repertoire of OSCC cell lines to better reflect the tumor heterogeneity that is under-represented by those currently available. Well-represented panel of cell lines have significant translational implications with respect to the preclinical evaluation of emerging therapeutic modalities for their effectiveness in relation to specific genetic background. Furthermore, together with recently well-characterized HNSCC cell lines [7, 8], the ORL cell line panel reported in this study, can enhance the development of molecular biomarkers that have hitherto been limited even in large scale studies of drug sensitivity [9, 10]. In conclusion, the availability of OSCC cell lines that reflect the genetic alterations in OSCC and those that respond differentially to different treatment modalities affords an opportunity to facilitate personalized care efforts that are currently actively being pursued [10, 33].

## MATERIALS AND METHODS

### Cell culture

Informed consent was obtained before collection of tissue specimens, and this study was approved by the Medical Ethics Committee, Faculty of Dentistry, University of Malaya (DPOP0306/0018/L). Sixteen OSCC cell lines were established from surgically resected OSCC tissue specimens as described previously [15]. Tissues were collected inα-MEM containing 20% (v/v) FBS, 200 iu/l penicillin, 200 μg/ml streptomycin and 0.1 μg/ml of fungizone. In the laboratory, tissues were washed in absolute ethanol for 20–30 seconds and then washed twice with phosphate-buffered saline (PBS) under sterile conditions. Tissues were minced, washed twice in culture media and re-suspended in α-MEM containing 20% (v/v) FBS, 200 iu/l penicillin, 200 μg/ml streptomycin, 0.4 ng/ml EGF, 2 μg/ml hydrocortisone and 2 mM L-glutamine, and seeded into 60 mm tissue culture dishes. Established cultures were cultured in Dulbecco's Modified Eagle Medium (DMEM)/F12 (1:1) supplemented with 10% (v/v) heat-inactivated fetal calf serum, 100 IU Penicillin/Streptomycin and 0.5 μg/ml hydrocortisone. All cultures were incubated in a humidified atmosphere of 5% CO_2_ at 37°C. Normal oral keratinocytes (NOK), ORL-232, ORL-235, and ORL-231 were derived from gingival tissues obtained during wisdom tooth extraction. A431 was obtained from American Type Culture Collection (ATCC, Manassas, VA) and were maintained in Dulbecco's Modified Eagle Medium (DMEM)/F12 (1:1) supplemented with 10% (v/v) heat-inactivated fetal calf serum, 100 IU Penicillin/Streptomycin and 0.5 μg/ml hydrocortisone. NOK were cultured in keratinocyte serum free media (KSFM; GIBCO, Carlsbad, CA, USA) supplemented with 25 μg/ml bovine pituitary extract, 0.2 ng/ml epidermal growth factor, 0.031 mM calcium chloride and 100 IU Penicillin/Streptomycin (GIBCO, Carlsbad, CA, USA). All cell lines were maintained in a humidified atmosphere of 5% CO_2_ at 37°C. Fibroblast contamination was routinely removed from ORL cell lines by trypsinization (0.25% trypsin/0.09% EDTA). All NOK were kept below 5 passages. Cell lines were routinely tested for presence of mycoplasma with MycoAlert mycoplasma detection kit (Lonza, Basel, Switzerland). Contaminated cultures were treated with plasmocin (Invivogen, San Diego, CA) according to manufacturer's protocol. Mycoplasma free lines were used for all experimentation.

### Cell line authentication by short tandem repeat genotyping

The ORL lines were authenticated to tumor or blood genomic DNA (gDNA) obtained from the matched respective donors. Briefly, polymerase chain reaction (PCR) was used to amplify fifteen short tandem repeat (STR) loci in the AmpFlSTR Identifiler^â^ PCR Amplification Kit (Applied Biosystems, Foster City, CA) or nine STR loci in the Promega StemElite kit (Promega, USA), both kits included a gender determination marker, Amelogenin. The PCR product was electrophoresed on an ABI Prism^®^ 3730xl Genetic Analyzer using a GeneScan^™^ 500LIZ^®^ and analyzed using GeneMapper^®^ v4.0 software (Applied Biosystems, Foster City, CA, USA). DNA profiles between the cell line and donor DNA were compared according to the recommendations from the International Cell Line Authentication Committee (ICLAC) guidelines [62].

### TRAPeze assay

Telomerase activity of ORL lines and NOK were determined using the TRAPeze RT Telomerase Detection kit S7710 (EMD Millipore, Billerica, MA) following instructions by the manufacturer. In brief, cells at 70^−^80% confluence were washed in PBS and lysed at 4°C in CHAPS lysis buffer. Cell debris was removed by centrifugation at 13,000 rpm for 20 min at 4°C and protein was quantified using bicinchoninic acid assay (BCA; Pierce, Rockford, IL, USA). A total of 1 μg protein was used for TRAPeze assay and telomerase activity was quantified on ABI 7500 Real-time PCR system (Applied Biosystems, Foster City, CA, USA).

### Cell proliferation and population doubling time

Proliferation curves from ORL cell lines and NOK cells were generated using the xCELLigence RTCA SP system (Roche Applied Science, Upper Bavaria, Germany). Cell proliferation was monitored every 15 minutes for the initial 2 hours and then every 30 minutes for the subsequent 120^−^166 hours. The start and end time during the log-growth phase of each cell lines were selected and doubling time was calculated by RTCA software (Roche Applied Science, Upper Bavaria, Germany). Doubling time was calculated from 2–3 independent experiments, with at least triplicate wells per experiment.

### Human papilloma virus (HPV) testing

DNA was extracted from cell lines grown to 70% confluence using the QIAamp DNA Mini kit (Qiagen, USA). The DNA samples were then amplified for the detection of HPV DNA presence using the HPV GenoArray DNA Test, a PCR-based HPV genotyping assay. This assay utilizes the L1 consensus primers to simultaneously amplify 21 HPV genotypes followed by flow through hybridization with immobilized genotype-specific probes (Hybribio Ltd., Hong Kong) [63]. Results indicative of HPV presence was interpreted according to the manufacturer's instructions.

### Animal care

All animal studies were done in accordance with a protocol approved by the Animal Ethics Committee of National University of Malaysia (CARIF/2011/CHEONG/22-MARCH/365-MAY-2011-MAY-2014). Female NU/NU mice and NOD/SCID mice of 4–6 weeks old were purchased from BioLASCO Co. Ltd. (Taipei, Taiwan). Mice were housed in appropriate sterile filter-capped cages, fed and watered *ad libitum*. Mice were monitored every other day for general behavioral abnormalities, signs of illness or discomfort, and any pathological changes were documented.

### Subcutaneous flank model

We examined the tumorigenicity of seven ORL cell lines in the subcutaneous flank model (ORL-48, ORL-115, ORL-136, ORL-150, ORL-174, ORL-188, ORL-204). These ORL cell lines were derived from patients with diverse risk habits and represented cell lines of the 3 different gene expression clusters (described below). A minimum of five NU/NU mice were used for each cell line. The ORL cell lines were resuspended at a concentration of 2 × 10^6^ cells in 200 μl DMEM-F12 serum-free media and were subcutaneously injected into the flanks of NU/NU mice. Mice were examined twice weekly for tumor development. Tumor size was measured using a digital caliper and tumor volume was determined using the formula LW^2^/2; whereby L and W represent length and the width of the tumor respectively. Tumor volume doubling time was calculated using the formula: ln2 × (t2 − t1)/ln[V(t2)/V(t1)]; whereby Vt1 is the tumor volume at time t1 and Vt2 is the tumor volume at time t2. Mice were euthanized on day 80 or earlier (if there were signs of illness or discomfort), and tumors on the flanks for each mouse were retrieved. The resected tumors were fixed in 4% formalin overnight and then transferred to 70% alcohol and processed for paraffin embedding for histopathological evaluation by pathologists (AM, TGK). Images of hematoxylin and eosin stained slides were acquired with the OlyVIA imaging software version 2.4 (Olympus, USA).

### Orthotopic tongue model

The tumorigenicity of the seven ORL cell lines mentioned above was also examined by the orthotopic tongue model. The orthotopic tongue model was established as reported previously [64]. Briefly, the ORL cell lines were injected into the posterior tongue at a concentration of 1 × 10^5^ in DMEM-F12 serum-free medium. Mice were examined twice weekly for tumor development in the tongue under anesthetic condition using isoflurane. Tumor measurement was given visually by the same operator for the duration of the study. Mice were euthanized after 40 days, the neck area of each mouse was carefully dissected to retrieve 4 to 5 cervical lymph nodes, and tongues for each mouse were also retrieved. The resected tissues were fixed in 4% formalin overnight and then transferred to 70% alcohol and processed for paraffin embedding for histopathological evaluation by pathologists (AM and TGK). Hematoxylin and eosin stained slides were acquired with the OlyVIA imaging software version 2.4 (Olympus, USA).

### DNA purification and copy number alterations

ORL cell lines and NOK were harvested by trypsinization at 70–80% confluence and DNA were extracted using the QIAamp DNA Mini kit (Qiagen, USA) according to manufacturer's instructions. Copy number alterations (CNA) analysis was performed on the Genome Wide Human Cytoscan HD array (Affymetrix, Santa Clara, CA). Data was analyzed with Chromosome Analysis Suite v2.0 (CHAS) and R software. Detailed analysis steps are included in Supplementary Methods.

### RNA purification and RNAseq library generation

ORL cell lines and NOK were lysed with TRI Reagent (Ambion, Carlsbad, CA, USA) at 70–80% confluence and extracted according to manufacturer's instructions. Libraries were constructed using 1 μg total RNA following Illumina TruSeq RNA Sample Preparation v2 Guide and 100 base pair paired-end sequencing was conducted as previously described [7].

### RNAseq data processing and OSCC subgroup detection

Sequencing reads were mapped to human reference genome (Ensembl release GRCh37) using Tophat2 version 2.0.9 with default parameters [65]. Variant calling was conducted with the use of GATK HaplotypeUnityper version 2.8 [66]. A series of variant calling and filtering criteria was applied to shortlist high confidence variants and identify potential somatic mutations (Supplementary Methods). Gene expression in Fragments Per Kilobase of exon per Million fragments mapped (FPKM) was extracted through the tuxedo protocol with the use of Cufflink (version 2.1.1) and Cuffdiff (version 2.2.0) [65]. For subgroup detection, genes with zero expression value in at least one sample were excluded and the raw FPKM values of remaining genes were log2 transformed. Unsupervised hierarchical clustering analysis was conducted on the most variably expressed genes determined by genes with median absolute deviation ≥ 0.5 (*n* = 7,053 genes) in the R environment program (R version 3.0.2) using the R package Consensus Cluster Plus [24]. The optimum cluster number (k) inferred from Consensus Cluster Plus was confirmed by the use of independent tools as described in Supplementary Methods. Pathway analysis was conducted with the use of Gene Set Enrichment Analysis (GSEA) [25] as described in Supplementary Methods.

### Molecular analysis of cell lines and validation of RNAseq data

Validation of *TP53* and *CDKN2A* mutations were conducted by Sanger sequencing on DNA extracted from donor tissue where available, while expression of *EGFR* was confirmed by western blotting. Details of these experiments are included in Supplementary Methods.

### Drug sensitivity assay

To determine whether gene expression profiles influence response to therapeutic drugs, we treated a sub-set of ORL cell lines from different gene expression clusters with DNA damaging agents that are routinely used in the clinic and cell viability was evaluated using 3-(4,5-dimethylthiazol-2-yl)-2,5-diphenyltetrazolium bromide (MTT) assay. Four cell lines each from Cluster 1 (ORL-48, ORL-156, ORL-150, ORL-204) and cluster 2 (ORL-115, ORL-153, ORL-207, ORL-214) were used. Briefly, 3000 cells in 80 μl culture medium per well was seeded in 96-well plates. After 16 hours incubation, the drugs which were diluted in 20 μl of culture medium were added into each well. Irinotecan hydrochloride (Sigma-Aldrich, USA; dissolved in DMSO), cisplatin (Sigma-Aldrich, USA; dissolved in phosphate buffer saline) and topotecan hydrochloride (Selleck Chemical, USA; dissolved in DMSO) were tested at concentrations ranging from 0.4–100 μM, whereas mitomycin C (Sigma-Aldrich, USA; dissolved in dH_2_O) was tested at concentrations ranging from 0.1 to 25 μM. Culture medium containing 0.01% (v/v) DMSO was used as vehicle control. Cells were treated for 48 hours and evaluations were done with six independent replicates for each drug. At the end of the treatment period, 20 μl of MTT solution (5 mg/ml) was added to each well and incubated for 4 hours at 37°C. The media was then removed and 100 μl of DMSO was added to solubilize the formazan crystal. The optical density was recorded by Synergy H1M microplate reader (BioTek Instruments, USA) at 570 nm. Five parameter logistic (5-PL) dose response curves were plotted using SoftMax Pro 5.4.5 (Molecular Devises, USA) and the 50 percent inhibitory concentration (IC_50_) of each drug of was obtained from the curve. Statistical differences were assessed by the Student's *t*-test. *p* < 0.05 was considered to be statistically significant.

Three cell lines each from Cluster 1 (ORL-48, ORL-150 and ORL-204) and cluster 2 (ORL-115, ORL-207 and ORL-214) were used to examine the effects of CDK1 inhibitor, RO3306. Cells were seeded on cover slips overnight and treated at concentrations ranging from 0–7 μM, for 24 hours. Following this, cell proliferation was evaluated by 5-ethynyl-2′-deoxyuridine incorporation using Click-iT EdU Cell Proliferation Assay Kit (Invitrogen, Carlsbad, CA, USA) according to manufacturer's protocol. Briefly, cells were incubated with 10 μM EdU for 2–6 hours prior to fixation with 3.7% formalin. The cells were permeabilized with 0.1% Triton X-100 in phosphate buffer, followed by EdU detection via a copper-catalyzed reaction and nuclei staining by Hoechst 33342 (Invitrogen, Carlsbad, CA, USA). The cover slips were then mounted on glass slides by using VECTASHIELD^®^ Mounting Medium (Vector Laboratories, Burlingame, CA, USA) and examined on an upright Olympus IX71 microscope (Olympus, Japan) with double bandpass filters to detect fluorescent-stained nuclei (DAPI: excitation 360–370 nm and emission 420 nm; Alexa 647: excitation 650 nm and emission 667 nm). Images were captured from 3–10 different fields of each treatment concentration and further analyzed with EBImage [67]. The percentage of EdU-labelled cells which indicates DNA-synthesizing cells was expressed as the percentage of red fluorescent nuclei over the total number cells reflected by DAPI-stained nuclei. Student's *T*-test was used to determine statistical significance between the percentages of EdU positive cells in each treatment concentration relative to untreated control cells. *P* value less than 0.05 was considered statistically significant.

## SUPPLEMENTARY MATERIALS FIGURES AND TABLES






