# Supplementary material for: Genetically-defined novel oral squamous cell carcinoma cell lines for the development of molecular therapies
Source: Oncotarget. 2016 Apr 1;7(19):27802–18. doi: 10.18632/oncotarget.8533 (PMC5053689; doi:10.18632/oncotarget.8533)
Supplement: Supplementary file 1 [file oncotarget-07-27802-s001.pdf]

Genetically-defined novel oral squamous cell carcinoma cell lines for the development of molecular therapies

Supplementary Materials

SUPPLEMENTARY METHODS

Copy number alterations

Copy number alterations (CNA) analysis was performed on the Genome Wide Human Cytoscan HD array (Affymetrix, Santa Clara, CA) as in the manufacturer's protocol using gDNA extracted with the QIAamp DNA Mini kit (Qiagen, USA) from ORL cell lines and normal keratinocyte (NOK) cell lines. Data was analyzed with Chromosome Analysis Suite v2.0 (CHAS) software NetAffx 32.3 (human genome build 19). In CHAS analysis, the generated data was normalized to 284 HapMap samples and 96 blood DNA samples from phenotypically healthy male and female individuals. Threshold of $\log_2$ ratio ≥ 0.203 was used to categorize the altered region as a copy number gain while $\log_2$ ratio ≤ -0.299 was considered a copy number loss. In CNA analysis, regions with at least 50 consecutive markers and $\geq$ 400 kbp in length were considered as potential CNA. Only tumor cell line specific alterations were reported.

Variant calling, filtering and identification of somatic mutations

Picard tools version 1.79 (http://picard.sourceforge.net) was used to remove duplicates reads from the aligned BAM file. Realignment and recalibration with Genome Analysis Tool Kit (GATK, version 2.8) [1] was conducted before variant calling. Variants were called using GATK HaplotypeUnityper and a series of filtering criteria was applied based on previous publications to distinguish true variant from false-positive or false-negative variants [2–4]. The following criteria has to be met to consider a variant true-positive;

1) Phred base quality and alignment read quality value ≥ 30
2) Variants in repetitive region of the genome and known RNA-editing site was discarded
3) Each variant position has to be sequenced with $\geq 10\times$ coverage.
4) Variants in located in SNP cluster (defined region with two or more mutation in 10bp window) were excluded.

Finally, variants were annotated with ANNOVAR [5] and Oncotator-web version v0.4.1.8 (http://www.broadinstitute.org/oncotator).

Identification of potential somatic mutation in the absence of matching normal cell line revolves around a filtering strategy based on removal of known polymorphism recorded in publicly available database and the removal of variants detected in the three normal human oral keratinocytes (NOK). In brief, previously recorded known variants [6], variants recorded in dbSNP135, dbSNP137, 6500exome, 1000 genome and complete genomics 69 (above 0.1% frequency) were excluded. Variants identified in the three NOK and all synonymous variants were also excluded.

Global transition and transversion frequencies across all the cell lines were calculated from SNP detected by RNA-seq (derived from Supplementary Table S4) and the proportion of transitions and transversions against all mutations across the cell lines were determined. The cell lines were grouped based on the association with risk habits and the proportion of transitions and transversions against all mutations, were compared. Base change of A > G and C > T (or vice versa) were classed as transition, while base change of G > T, A > C, T > A and C > G (or vice versa) were classed as transversion.

Validation of subgroup discovery

The most optimal number of cluster (k = 3) was determined by inspection of consensus cluster plus results and principal component analysis plot. Statistical assessment of k = 3 was conducted by performing all pairwise comparison between subgroup using SigClust [7]. In addition silhouette analysis [8] was used to further validate if ORL cell lines were assigned to their appropriate cluster.

Pathway analysis

Pathway analysis was conducted using the Gene Set Enrichment Analysis (GSEA) software [9] version 2.1.0. Kyoto Encyclopedia of Genes and Genome (KEGG) [10] gene set database version 4.0 was used to determine enriched pathways in each subgroup. Subgroup comparison was conducted by comparing one subgroup against all other groups generating three degree of comparison (Subgroup 1 v Subgroup 2, Subgroup 1 v Subgroup 3 and Subgroup 2 v Subgroup 3). Only genes with > 0.2 FPKM in at least 50% of the samples were used as input for GSEA program. Enrichment score was calculated based on the weighted enrichment statistics. Genes were ranked based on Signal2Noise ratio and false discovery rate was estimated by gene set permutation over 1,000 runs.

Sanger sequencing of *TP53* and *CDKN2A*

Characterization of *TP53* mutations have been previously described elsewhere [11, 12]. Mutation status of *CDKN2A* was characterized as follows; genomic DNA (gDNA) from cell pellets were extracted by QIAamp DNA Mini kit (Qiagen, USA). Tumor gDNA from patients from which cell lines were derived were obtained from the Malaysian Oral Cancer Database & Tissue Bank System (MOCDTBS) [13]. Exon 1α, 1β, exon 2 and exon 3 of *CDKN2A* gene were amplified by polymerase chain reaction (PCR) on the GeneAmp PCR system 9700 (Applied Biosystems, CA, USA). Primer designs were as described previously by others [14], with some modifications: exon 1α, sense 5′ GGTCCCAGTCTGCAGTTAAG 3′; exon 2, sense 5′ AGCTTCCTTTCCGTCATGC 3′ and antisense 3′ GGAAGCTCTCAGGGTACAAATTC 3′. PCR was performed in a 50 µl reaction mixture containing 1.5 mM $MgCl_2$, 0.2 mM dNTPs, 10 µM primer pairs, 1.25 U GoTaq® DNA polymerase (Promega, Madison, WI), with cycling conditions: 94ºC (2 min), followed by 40 cycles of 94ºC (30 sec), 58°C−67ºC annealing (30 sec), 72ºC (30 sec) and final extension at 72ºC for 7 min. Results were further verified by an independent experiment in a 50 µl PCR reaction containing 10× buffer, 25 mM $MgSO_4$, 2 mM dNTPs, 10 µM primer pairs, 2.5 U KOD Hot Start DNA polymerase (EMD Millipore, Billerica, MA). PCR cycling conditions were as described above. In both PCR reactions, 200 ng of cell line gDNA and 25−130 ng of tumor gDNA were used. PCR amplicons were visualized in a 2% (w/v) agarose gel stained with ethidium bromide before being purified with QIAquick PCR purification kit (Qiagen, USA) and sequenced as described previously [12].

Immunobloting

Total protein was extracted in RIPA lysis buffer (0.5% NaDOC, 0.1% SDS, 25 mM HEPES pH 7.5, 0.3 M NaCl, 1.5 mM $MgCl_2$, 0.2 mM EDTA, 1% Triton X-100, 20 mM β-glycerophosphate, 0.1 mM Na_3VO_4, 0.5 mM DTT, 1:100 Halt Protease inhibitor cocktail (Pierce Biotechnology, IL, USA) and 60 µg protein was loaded into each lane of a 12% sodium dodecyl sulfate-polyacrylamide gel. Following electrophoresis, proteins were transferred onto PVDF membranes. Membranes were immunoblotted with antibodies for total EGFR (ab2430, 1:200; Abcam, Cambridge, MA). Blots were washed and incubated with horseradish peroxidase-conjugated secondary antibodies (1:7,500–10,000; Southern Biotech, Birmingham, AL). Blots were developed using enhanced chemiluminescence method and imaged using ChemiImager™ Imaging Systems (Alpha Innotech, San Leandro, CA). Membranes were reprobed with anti-actin monoclonal antibody (C4, 1:200; EMD Millipore) to control for loading variations.

Statistical analysis

Instat and Prism software (GraphPad Software Inc., San Diego, CA) was used for statistical analyses and graph design, respectively. For comparison of two groups, two-tailed, unpaired *t*-test with Welch correction was performed. Multiple group comparisons were performed via one-way ANOVA, with accompanying Tukey-Kramer post test if $p < 0.05$. All bar graphs are presented as mean ± standard deviation (SD). Numbers of independent experiments are indicated by the N value. Significant differences ($p < 0.05$) are denoted in figures by asterisks (*).

REFERENCES

1. DePristo MA, Banks E, Poplin R, Garimella KV, Maguire JR, Hartl C, Philippakis AA, del Angel G, Rivas MA, Hanna M, McKenna A, Fennell TJ, Kernytsky AM, et al. A framework for variation discovery and genotyping using next-generation DNA sequencing data. Nat Genet. 2011; 43:491–498.
2. Horvath A, Pakala SB, Mudvari P, Reddy SDN, Ohshiro K, Casimiro S, Pires R, Fuqua SAW, Toi M, Costa L, Nair SS, Sukumar S, Kumar R. Novel Insights into Breast Cancer Genetic Variance through RNA Sci Rep. 2013; 3.
3. Piskol R, Ramaswami G, Li JB. Reliable Identification of Genomic Variants from RNA-Seq Data. Am J Hum Genet. 2013; 93:641–651.
4. Quinn EM, Cormican P, Kenny EM, Hill M, Anney R, Gill M, Corvin AP, Morris DW. Development of Strategies for SNP Detection in RNA-Seq Data: Application to Lymphoblastoid Cell Lines and Evaluation Using 1000 Genomes Data. PloS One. 2013; 8.
5. Wang K, Li M, Hakonarson H. ANNOVAR: functional annotation of genetic variants from high-throughput sequencing data. Nucleic Acids Res. 2010; 38:e164.
6. Glusman G, Caballero J, Mauldin DE, Hood L, Roach JC. Kaviar: an accessible system for testing SNV novelty. Bioinformatics. 2011; 27:3216–3217.
7. Liu Y, Hayes DN, Nobel A, Marron JS. Statistical Significance of Clustering for High-Dimension, Low-Sample Size Data. J Amer Statist Assoc. 2008; 103:1281–1293.
8. Rousseeuw PJ. Silhouettes - a graphical aid to the interpretation and validation of cluster-analysis. J Comput Appl Math. 1987; 20:53–65.
9. Subramanian A, Tamayo P, Mootha VK, Mukherjee S, Ebert BL, Gillette MA, Paulovich A, Pomeroy SL, Golub TR, Lander ES, Mesirov JP. Gene set enrichment analysis: a knowledge-based approach for interpreting genome-wide expression profiles. Proc Natl Acad Sci U S A. 2005; 102:15545–15550.
10. Kanehisa M, Goto S. KEGG: kyoto encyclopedia of genes and genomes. Nucleic Acids Res. 2000; 28:27–30.

11. Zanaruddin SN, Yee PS, Hor SY, Kong YH, Ghani WM, Mustafa WM, Zain RB, Prime SS, Rahman ZA, Cheong SC. Common oncogenic mutations are infrequent in oral squamous cell carcinoma of Asian origin. PLoS One. 2013; 8:e80229.
12. Hamid S, Lim KP, Zain RB, Ismail SM, Lau SH, Mustafa WM, Abraham MT, Nam NA, Teo SH, Cheong SC. Establishment and characterization of Asian oral cancer cell lines as *in vitro* models to study a disease prevalent in Asia. Int J Mol Med. 2007; 19:453–460.
13. Zain R, Athirajan V, Ghani W, Razak I, Raja Latifah R, Ismail S, Sallam A, Bustam A, Rahman Z, Hussien A, Talib N, Cheong S, Jallaludin A. An oral cancer biobank initiative: a platform for multidisciplinary research in a developing country. Cell Tissue Bank. 2012:1–8.
14. Kubo Y, Urano Y, Matsumoto K, Ahsan K, Arase S. Mutations of the *INK4a* Locus in Squamous Cell Carcinomas of Human Skin. Biochem Biophys Res Commun. 1997; 232:38–41.

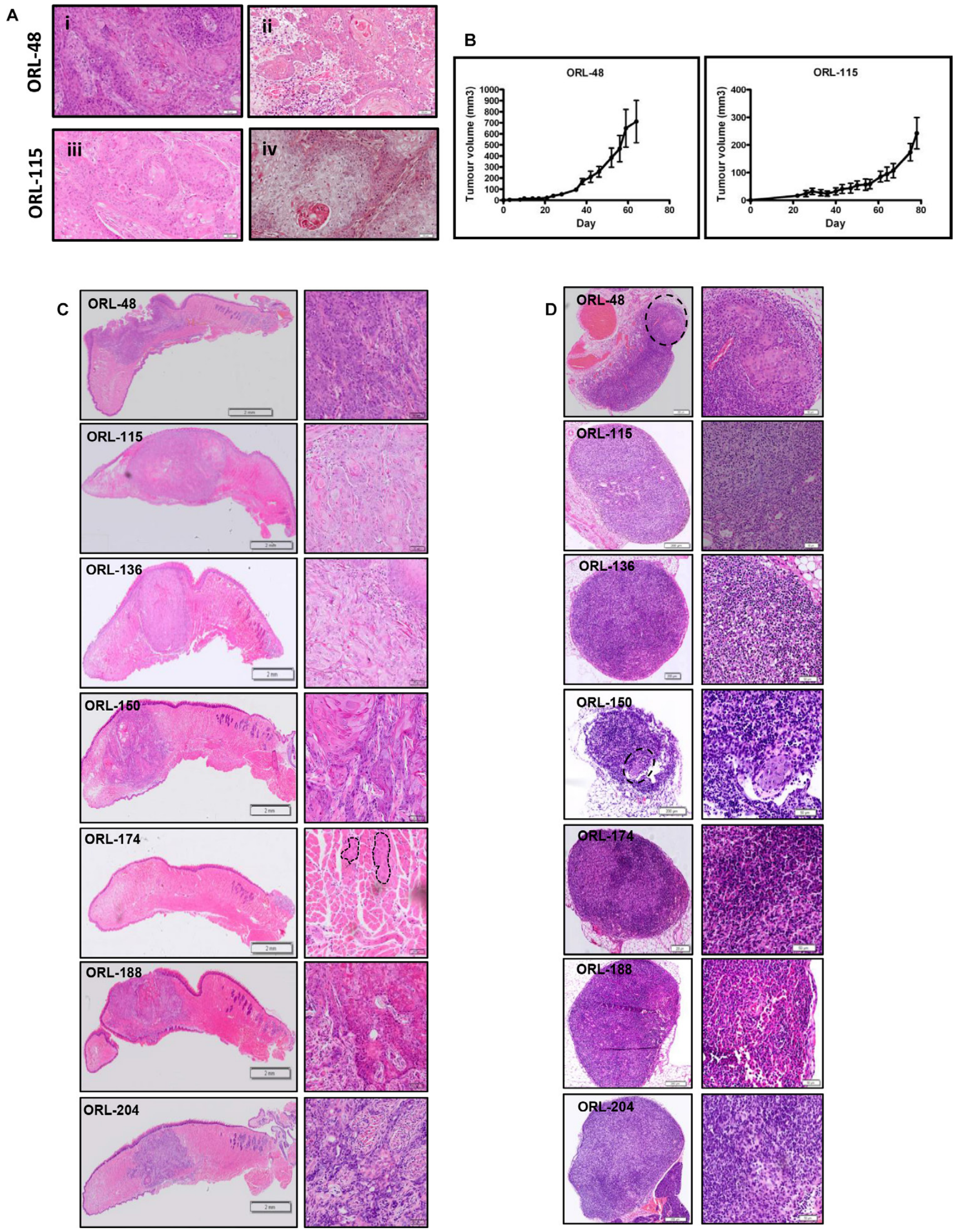

Supplementary Figure S1: H&E staining of tumor xenograft. (**A**) ORL-48 and ORL-115 cell lines formed subcutaneous tumors in Nu/Nu mice (i & iii) that have similar histology to the tumor tissue of the respective patients (ii & iv). Insets are of 200× magnification of an area of the tumor. (**B**) Subcutaneous tumor growth of ORL-48 and ORL-115 on NOD/SCID mouse. (**C**) Hematoxylin and eosin staining of tongue tumors. Right panel is of 100× magnification of an area within the tumor. (**D**) Hematoxylin and eosin staining of lymph nodes from the orthotopic tongue tumors. Only ORL-48 and ORL-150 showed presence of metastatic tumor cells in the lymph nodes. Right panel is of 100× magnification of the selected area.

A

B

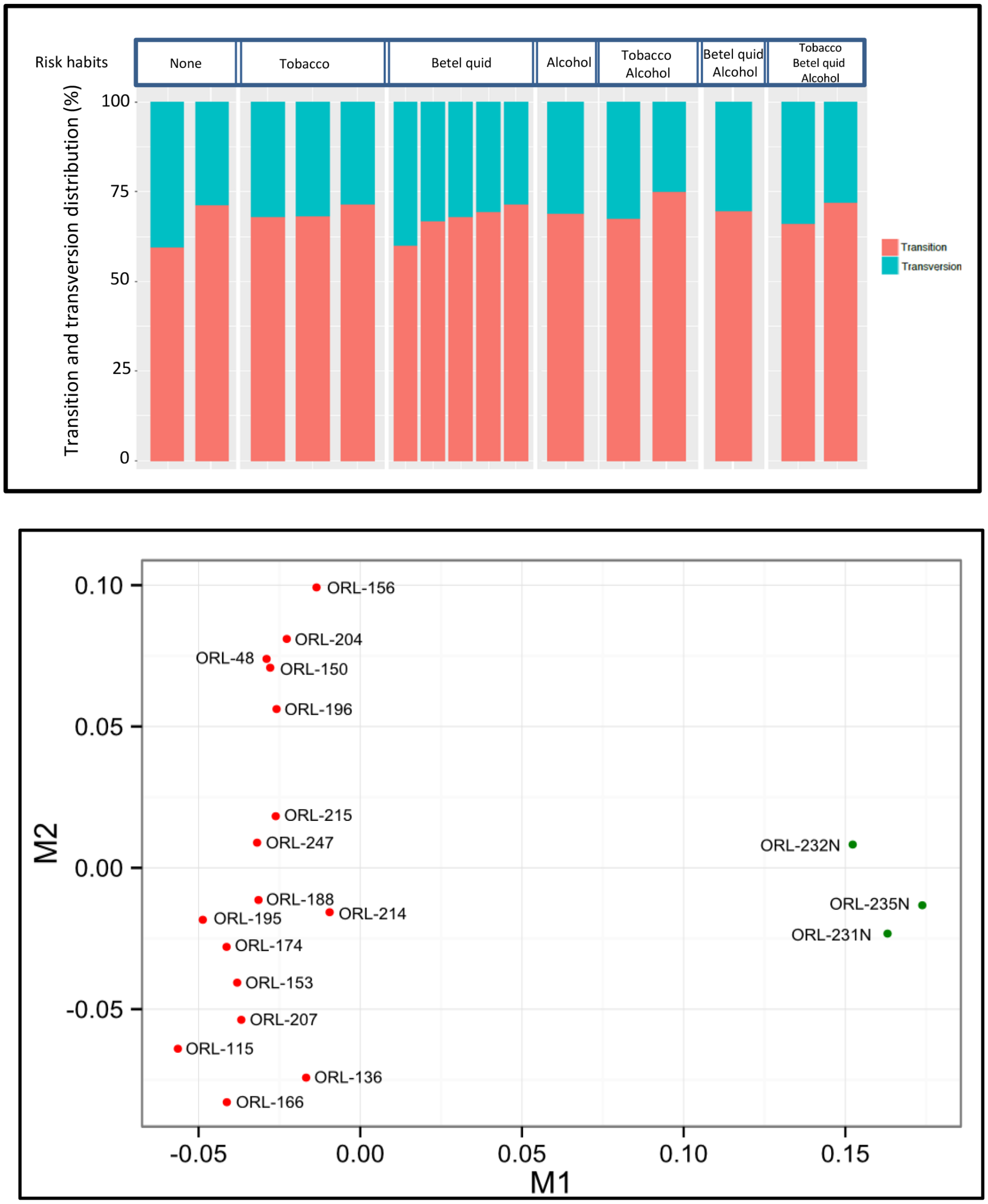

Supplementary Figure S2: (**A**) Distribution of transition and transversion mutations in the cell lines associated with different risk habits. (**B**) Multi-dimensional scaling plot of gene expression in each ORL cell line. Global gene expression profiling unambiguously distinguish normal oral keratinocyte cultures (green) from tumor cell lines (red). M1 and M2 represent distance measure designed to show the relationship between samples.

A

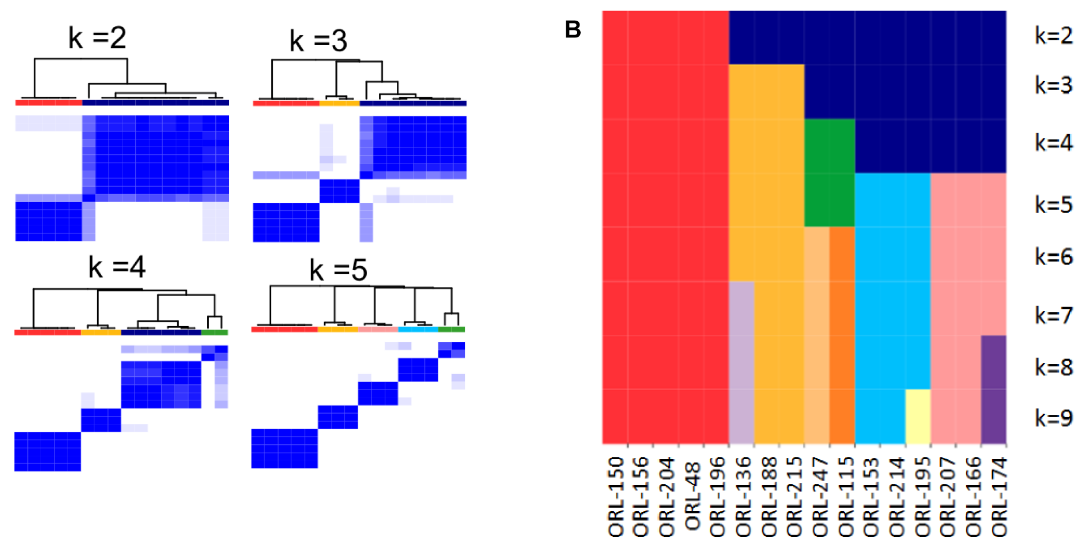

C

	Subgroup 1	Subgroup 2	Subgroup 3
Subgroup 1			
Subgroup 2	1.42E-26		
Subgroup 3	8.92E-43	1.07E-02	

D

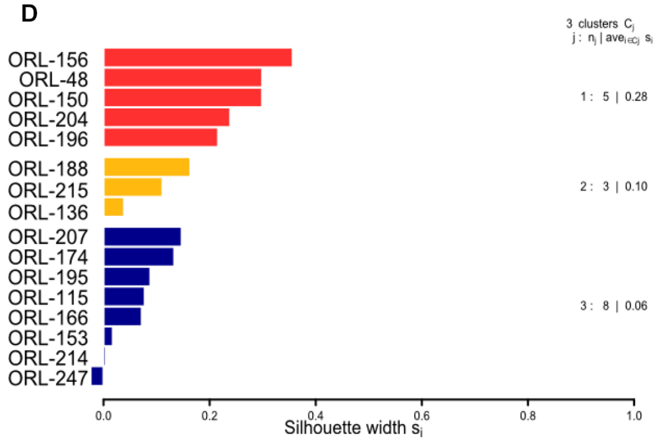

E

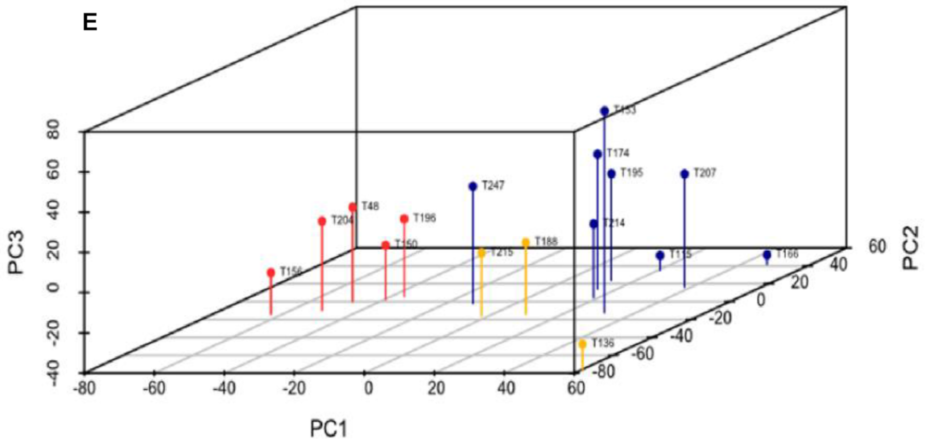

Supplementary Figure S3: Validation of unsupervised hierarchical clustering by consensus cluster. (**A**) Consensus cluster matrices for $k = 2–4$. The intensity of each blue square represents the proportion of time the sample falls in the same cluster over 1,000 iterations. Samples that do not occur in the same cluster are colored by a white pixel. (**B**) Tracking plot of each sample across different cluster size. Samples that maintain same grouping (same color across different k) are indicative of robust cluster. Samples which change cluster across different k are considered unstable members of a group. Transition from $k = 3$ to $k = 4$ indicates changes of only 2 samples. (**C**) Comparison of each subtype in relation to each other showed statistical significant by SigClust. (**D**) Silhouette plot showed all, except one sample (ORL-247) are well represented within assigned subtype. (**E**) Principal component analysis of the most variable expressed genes (Red = Cluster 1, Blue = Cluster 2 and Yellow = Cluster 3) supports observation of 3 clusters.

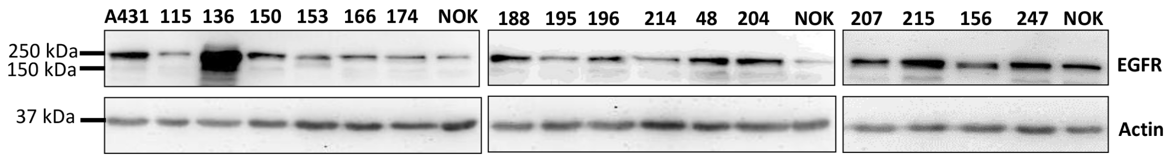

Supplementary Figure S4: Levels of EGFR protein in ORL-lines. Protein levels of EGFR was detected by western blotting and compared to the levels detected in normal oral keratinocytes. A431 is included as a positive control for EGFR expression. NOK: Normal oral keratinocytes.

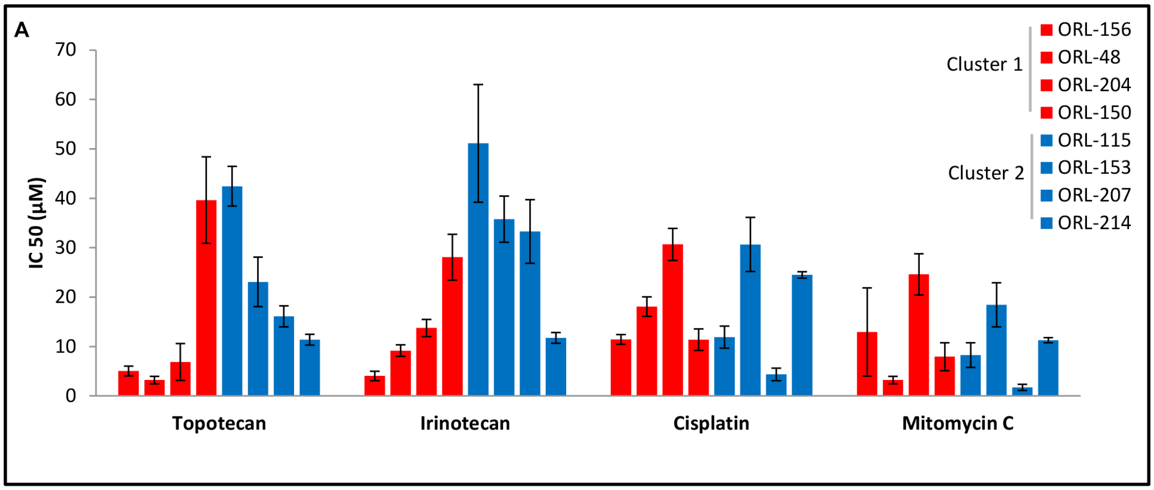

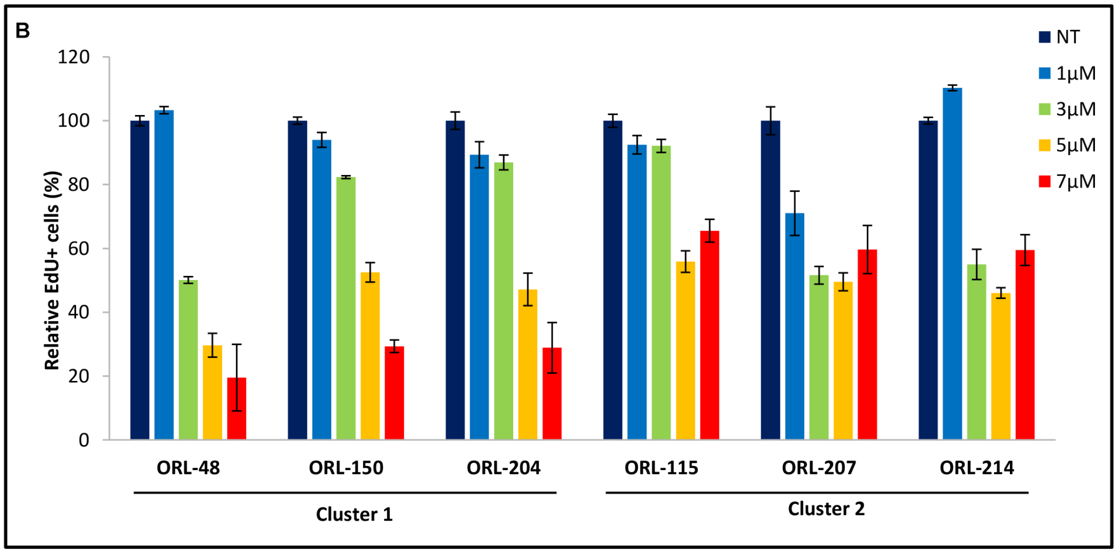

Supplementary Figure S5: Cell lines from different gene expression clusters have differential response to specific drugs. (**A**) IC_{50} of each cell lines in response to topotecan, irinotecan, cisplatin and mitomycin C. (**B**) ORL cell lines of from Cluster 1 are more sensitive to CDK1 inhibitor, RO-3306 compared to cell lines from Cluster 2 where cell proliferation was inhibited by more than 80% at 7 µM in Cluster 1 compared to less than 40% in cell lines from Cluster 2. The data is a representative of 3 independent experiments. *denotes significance of $p < 0.05$.

Supplementary Table S1: Authentication of ORL cell lines

Cell line	Gender[a]	Passage	Primary site	Sample	AMEL	CSF1PO	D13S317	D16S539	D18S51	D19S433	D21S11	D2S1338	D3S1358	D5S818	D7S820	D8S1179	FGA	TH01	TPOX	vWA	% Match*
ORL-48	F	25	Gingiva	Cell Line	X	10, 11	12	11, 13	14	13	29, 32.2	19, 25	15	11, 12	11	12, 15	24, 25	6, 9	11	19	98.0
				Matched tissue	X	10, 11	12	11, 13	14	13	29, 32.2	19, 25	15	11, 12	11	12, 15	24, 25	6, 9	11	17, 19	
ORL-115	F	29	Gingiva	Cell Line	X	11	8, 12	11	14	14	29, 30	18, 23	16	12	8, 10	10, 13	20, 23	9	9, 10	15, 16	100
				Matched Blood	X	11	8, 12	11	14	14	29, 30	18, 23	16	12	8, 10	10, 13	20, 23	9	9, 10	15, 16	
ORL-136	M	4	Tongue	Cell Line	X, Y	8, 11	8, 9	9, 12	14, 15	12, 16	29.2, 32.2	19, 22	15, 16	10	9, 12	14, 16	22	6, 9	9, 10	16, 18	98.4
				Matched Blood	X, Y	8, 11	8, 9	9, 12	14, 15	12, 16	29.2, 32.2	19, 22	15, 16	10	9, 12	14, 16	19, 22	6, 9	9, 10	16, 18	
ORL-153	M	21	Gingiva	Cell Line	X, Y	10	10, 14	12	13, 14	14, 16	28,31	19, 23	16, 17	11, 12	7, 12	10, 17	21	9, 9.3	11	17, 18	96.4
				Matched Blood	X, Y	10	10, 14	12	13, 14	14,16	28, 31	19, 23	16, 17	11, 12	7, 12	10, 18	21	9, 9.3	11	17, 18	
ORL-156	M	5	Tongue	Cell Line	X	9	9	12	NA	NA	28	NA	NA	11	10, 11	NA	NA	7, 8	8, 11	14, 16	87.5
				Matched tissue	X, Y	9, 11	9	12	NA	NA	28, 29	NA	NA	11, 13	10, 11	NA	NA	7, 8	8, 11	14, 16	
ORL-150	M	30	Tongue	Cell Line	X	12	12	11, 12	11	14, 15.2	29, 32.2	23	16	11, 13	9, 11	14, 16	19, 24	6, 9	11	14, 19	88.8
				Matched Blood	X, Y	12	11, 12	11, 12	11, 14	14, 15.2	29, 32.2	23	15, 16	11, 13	8, 11	14, 16	19, 24	6, 9	11	14, 19	
ORL-166	F	17	Tongue	Cell Line	X	12	10, 11	12	15	13, 15	30.2, 32.2	22, 23	15, 16	12	10, 11	13, 14	21, 24	9	8, 11	14, 18	98.0
				Matched Blood	X	12	10, 11	12	15	13, 15	30.2, 32.2	22, 23	15	12	10, 11	13, 14	21, 24	9	8, 11	14, 18	
ORL-174	F	19	Tongue	Cell Line	X	12	12, 13	9, 11	12, 19	14	28, 29	20	16	9	8, 10	11, 14	24, 25	8	8, 9	14, 18	92.6
				Matched tissue	X	11, 12	12, 13	9, 11	12, 19	14, 14.2	28, 29	20	16, 18	9, 12	8, 10	11, 14	24, 25	8	8, 9	14, 18	

ORL-188	M	20	Tongue	Cell Line	X, Y	9, 12	8, 11	10	17, 19	14, 14.2	31	19	16	10, 13	10, 11	13, 16	24.2, 27	7, 8	9	16	96.3
				Matched tissue	X, Y	9, 12	8,11	9, 10	17, 19	14, 14.2	31	19	16, 17	10, 13	10, 11	13, 16	24.2, 27	7, 8	9	16	
ORL-195	F	25	Buccal mucosa	Cell Line	X	11, 12	11, 12	11, 12	13	13, 15.2	29, 31.2	19	17	11, 16	10, 11	14, 16	24	6, 9	8, 9	16, 18	96.4
				Matched tissue	X	11, 12	11, 12	11, 12	13	13, 15.2	29, 31.2	19	16, 17	11, 15, 16	10, 11	14, 16	24	6, 9	8, 9	16, 18	
ORL-196	F	7	Buccal mucosa	Cell Line	X	10, 12	12	12, 13	15	14.2, 16	29, 32.2	22, 23	14, 17	13, 14	8	13, 15	21, 22	6, 7	8, 11	14, 16	94.6
				Matched Blood	X	10, 12	12	12, 13	15, 16	14.2, 16	29, 32.2	22, 23	17	13, 14	8	13, 15	22	6, 7	8, 11	14, 16	
ORL-204	M	25	Buccal mucosa	Cell Line	X, Y	12	8, 9	9	16	11, 13.2	32, 34.2	20, 21	15	12	12	15	17	6, 8	11	14, 16	95.8
				Matched tissue	X, Y	12	8, 9	9	16	11, 13.2	32, 34.2	20, 21	15, 17	12	12	15	17, 19	6, 8	11	14, 16	
ORL-207	F	13	Tongue	Cell Line	X	9	12	9, 13	12, 18	13, 15.2	30, 31.2	18, 23	15	12	8, 13	12, 16	22	9.3	11, 12	17	88.5
				Matched Blood	X	9, 12	9, 12	9, 13	12, 18	13, 15.2	30, 30.2	18, 23	15	12, 13	8, 13	12, 16	21, 22	9.3	11, 12	17	
ORL-214	F	6	Buccal mucosa	Cell Line	X	10, 12	10, 12	9, 11	13	12, 13	30, 33.2	19, 21	15, 16	11, 13	10	13, 16	23, 25	8, 9	8, 11	16, 18	98.2
				Matched Blood	X	10, 12	10, 12	9, 11	13	12, 13	30, 33.2	19, 21	15, 16	11, 13	10	13, 16	23, 25	8	8, 11	16, 18	
ORL-215	M	23	Tongue	Cell Line	X, Y	11, 12	8, 11	11	14, 18	12, 15.2	28, 29	19, 20	15, 18	11	10, 11	10, 16	22, 23	6	11	16, 19	100
				Matched Blood	X, Y	11, 12	8, 11	11	14, 18	12, 15.2	28, 29	19, 20	15, 18	11	10, 11	10, 16	22, 23	6	11	16, 19	
ORL-247	M	12	Tongue	Cell Line	X	10, 12	11, 12	10, 12	NA	NA	29, 32.2	NA	NA	12	11	NA	NA	6, 9.3	8	16, 17	90.9
				Matched tissue	X, Y	10, 12	12	10, 12	NA	NA	29, 32.2	NA	NA	12	11	NA	NA	6, 9.3	8, 11	16, 17	

Abbreviations: [a] M: Male, F: female.
*Percent match is calculated according to International Cell Line Authentication Committee (ICLAC) guidelines.

Supplementary Table S2: Relative hTERT activity of ORL lines

Cell lines	Relative hTERT activity
ORL-215, ORL-188, ORL-115, ORL-150	+++++
ORL-153, ORL-204, ORL-48	++++
ORL-196, ORL-156	+++
ORL-174, ORL-195, ORL-207, ORL-214, ORL-166, ORL-136, ORL-247	++
ORL-232, ORL-321, ORL-235	+/−

”+” denotes the levels of hTERT activity, the more “+”, the higher the hTERT activity in a cell line relative to one another.

Supplementary Table S3: Genomic region of recurrent copy number alterations (CNAs) in ORL lines

Supplementary Table S4: List of non-synonymous single nucleotide variants and indels in 16 oral cancer cell lines

Supplementary Table S5: Status of TP53 in ORL cell lines compared to available fresh frozen tissues

TP53					
**Cell Line**	**Mutation**	**Exon**	**Cell line**	**Matching fresh frozen tissue**	**Concordance between cell line & tissue**
ORL-115	c.336_338delCTT	4	√	NM	No
ORL-150	c. 414delC	5	√	√	Yes
ORL-153	NM	-	NM	NM	Yes
ORL-166	c.524G > A	5	√	√	Yes
ORL-174	c.916C > T	8	√	√	Yes
ORL-195	c.710T > A	7	√	√	Yes
	c.507delG	5	√	√	Yes
ORL-204	c.817C > T	8	√	√	Yes
ORL-214	c. 460G > A	5	√	√	Yes
	c.722C > T	7	√	√	Yes
ORL-215	c.844C > T	8	√	√	Yes
ORL-247	c.642_643delTA	6	√	√	Yes
NM: No Mutation				**Overall Concordance**	**11/12 (92%)**

Supplementary Table S6: Status of CDKN2A in ORL cell lines compared to available fresh frozen tissues

CDKN2A/p16					
**Cell Line**	**Mutation**	**Exon**	**Cell line**	**Matching fresh frozen tissue**	**Concordance between cell line & tissue**
ORL-115	c. 151-1G > C	2	√**	√*	Yes
ORL-136	NM		NM	NM	Yes
ORL-150	c.238C > T	2	√**	√*	Yes
ORL-153	c.238C > T	2	√**	√*	Yes
ORL-156	c.238C > T	2	√**	NM	No
ORL-174	c.262G > T	2	√*	√*	Yes
ORL-188	c.205G > T	2	√*	NM	No
ORL-195	Whole exon deletion	1	√**	NM	No
ORL-196	c.238C > T	2	√**	√*	Yes
ORL-204	c.238C > T	2	√**	√*	Yes
ORL-207	NM		NM	NM	Yes
ORL-214	NM	-	NM	NM	Yes
ORL-247	c. 151-1G > A	2	√**	√*	Yes
				Overall Concordance	10/13 (77%)

NM - No Mutation
* - Heterozygous.
** - Homozygous.

Supplementary Table S7: Top ten enriched pathways in each subgroup determined by gene set enrichment analysis (GSEA)

Cluster 1 [ORL-150, ORL-156, ORL-204, ORL-48, ORL-196]						
**PATHWAY NAME**	**SIZE**	**ES**	**NES**	**NOM *p*-value**	**FDR *q*-value**	**Enriched Genes**
DNA REPLICATION	32	0.809	2.806	0.00E + 00	0.00E + 00	MCM5,RFC2, SSBP1, MCM4, POLE, PRIM2, RFC5, RPA1, RFC4, RPA3, MCM2, RFC3, POLE3, POLD2, PCNA, MCM3, RNASEH2A, RNASEH2B, LIG1, POLA2, MCM7, MCM6, RNASEH2C, DNA2
MISMATCH REPAIR	21	0.811	2.564	0.00E + 00	0.00E + 00	RFC2, MLH1, MSH2, EXO1, SSBP1, MSH3, RFC5, RPA1, RFC4, RPA3, RFC3, POLD2, PCNA, LIG1, MSH6
SPLICEOSOME	98	0.591	2.559	0.00E + 00	0.00E + 00	LSM3, HNRNPM, SRSF1, SNRPF, U2AF1, NCBP1, PHF5A, TRA2B, PRPF4, RBM8A, SNRPD1, SNRPE, SF3A1, PPIL1, SNRPC, MAGOH, SRSF3, PRPF8, SNRNP40, RBM22, SRSF7, EFTUD2, LSM2, U2SURP, SF3B3, PRPF38A, SRSF9, PRPF19, WBP11, SART1, SNRPG, LSM6, LSM4, CDC40, RBM17, HNRNPC, SRSF10, DHX16, ACIN1, PRPF31, EIF4A3, SRSF6, LSM5, SF3B5, CWC15, PPIH, CDC5L, TRA2A, SNRPB2, TXNL4A, PQBP1, TCERG1, CTNNBL1, DDX23, THOC3, PRPF3, DDX42, NHP2L1, CCDC12, CRNKL1, PRPF38B, THOC1, BUD31, SRSF8, HNRNPA1, HSPA1A, PRPF18, USP39, SNRPA1
CELL CYCLE	105	0.569	2.546	0.00E + 00	0.00E + 00	MCM5, PKMYT1, CDC20, CDC25A, MCM4, CCNB1, CDK2, CCNE2, TTK, CDK4, ANAPC5, CDC26, CCNE1, MCM2, ORC6, TFDP1, RBL1, CDC7, PCNA, STAG1, ORC1, MCM3, MCM7, E2F1, MAD2L1, MCM6, ORC5, HDAC2, ATR, CCNH, E2F3, CDC27, CDC23, YWHAE, CDK6, CREBBP, PRKDC, CDC25C, CDC45, SKP2, BUB1, ESPL1, CUL1, CCNA1, DBF4, CDK1, ORC3, PTTG1, BUB3, YWHAH, MAD2L2, SMC3, ANAPC4, FZR1, MAD1L1, ANAPC13, ABL1
PROTEASOME	34	0.692	2.472	0.00E + 00	0.00E + 00	PSMD2, PSMC3, PSMD12, PSMC5, PSMB6, PSMB2, PSMA7, PSMD13, PSMD3, PSMD11, PSMB3, PSMB1, PSMB4, PSMB7, PSMD1, POMP, PSMB5, PSMD4, PSMC6, PSMC1, PSMD14, PSMD6, PSMA3, PSMC2
HOMOLOGOUS RECOMBINATION	24	0.761	2.442	0.00E + 00	0.00E + 00	RAD51C, RAD51, SSBP1, EME1, RAD54L, XRCC2, RPA1, BLM, BRCA2, RPA3, POLD2, TOP3A

NUCLEOTIDE EXCISION REPAIR	39	0.597	2.184	0.00E + 00	1.09E–04	RFC2, GTF2H3, POLE, RFC5, RPA1, RFC4, RPA3, RFC3, GTF2H1, POLE3, POLD2, PCNA, LIG1, CCNH, ERCC2, RAD23B, CUL4A, RAD23A, POLE4
OOCYTE MEIOSIS	74	0.489	2.033	0.00E + 00	5.82E–04	PKMYT1, CDC20, CCNB1, CDK2, CCNE2, ANAPC5, SGOL1, CDC26, CCNE1, PPP1CC, MAD2L1, CDC27, CDC23, YWHAE, CDC25C, PPP2R1B, BUB1, PPP2CB, ESPL1, CUL1, CDK1, PTTG1, YWHAH, MAD2L2, SMC3, AURKA, ANAPC4, ANAPC13, PPP2R5E, MAP2K1, CCNB2, YWHAB, ANAPC10, PRKACA, FBXO43
RNA DEGRADATION	48	0.536	2.004	0.00E + 00	1.05E–03	LSM3, EXOSC8, HSPD1, EXOSC2, ENO1, SKIV2L2, HSPA9, LSM2, EXOSC9, EXOSC1, RQCD1, PAPOLA, LSM6, LSM4, WDR61, PNPT1, LSM5, EXOSC10, ENO3, LSM1, CNOT7
BASAL TRANSCRIPTION FACTORS	24	0.610	1.973	0.00E + 00	1.27E–03	TAF5, GTF2H3, TAF9B, GTF2H1, TBP, GTF2F2, GTF2A1, TAF5L, TAF9, TAF6, TAF11, GTF2E1, TBPL1, GTF2F1, TAF13

Cluster 2 [ORL-247, ORL-115, ORL-153, ORL-214, ORL-195, ORL-207, ORL-166, ORL-174]						
**PATHWAY NAME**	**SIZE**	**ES**	**NES**	**NOM *p*-value**	**FDR *q*-value**	**Enriched Genes**
METABOLISM OF XENOBIOT-ICS BY CYTO-CHROME P450	26	0.681	2.396	0.00E + 00	0.00E + 00	ALDH3B2, EPHX1, MGST2, GSTK1, UG-T1A6, MGST1, CYP3A5, AKR1C2, GSTA4, AKR1C3, GSTT2, CYP2S1, ALDH3A1, AKR1C1
DRUG METABO-LISM CYTO-CHROME P450	24	0.657	2.294	0.00E + 00	0.00E + 00	ALDH3B2, MGST2, GSTK1, CYP2A6, UG-T1A6, MGST1, MAOA, CYP2D6, CYP3A5, GSTA4, GSTT2, ALDH3A1, FMO5
RETINOL ME-TABOLISM	19	0.702	2.235	0.00E + 00	3.55E−04	ALDH1A2, DHRS9, RETSAT, RDH10, PNPLA4, CYP2A6, UGT1A6, DGAT2, DHRS3, CYP3A5, RDH16, CYP26B1, DHRS4
ABC TRANS-PORTERS	20	0.688	2.230	0.00E + 00	2.66E−04	ABCA12, ABCA1, ABCC10, ABCG1, ABCA2, ABCA13, ABCD1, TAP1, ABCC5, ABCD4, ABCC3, ABCC2
STEROID HOR-MONE BIOSYN-THESIS	18	0.699	2.189	0.00E + 00	5.02E−04	SRD5A3, STS, SULT2B1, UGT1A6, CYP19A1, CYP7B1, SRD5A1, CYP3A5, AKR1C2, AKR1C3, AKR1C1
SYSTEMIC LU-PUS ERYTHEMA-TOSUS	43	0.543	2.185	0.00E + 00	4.18E−04	HIST2H2BE, HIST1H2AC, HIST1H2BF, HIST1H4K, HIST1H2BC, HIST1H4E, HIST1H4H, CD86, HIST3H2A, HIST1H2BG, HIST1H2AE, HIST1H4J, HIST4H4, HIST1H-2BK, C3, C5, HIST1H2AI, TRIM21, ACTN4, H2AFY2, HIST1H2AK, H2AFJ
PEROXISOME	54	0.470	1.972	0.00E + 00	4.49E−03	EPHX2, IDH1, PEX1, CROT, GSTK1, ACOX1, PEX13, ACSL1, ABCD1, PEX11A, ACSL5, SCP2, ABCD4, PHYH, PEX11G, CAT, DHRS4, IDH2, ACOT8, HMGCL, ACOX3, ACSL3, PEX6, PXMP4, PRDX5

LYSOSOME	91	0.399	1.860	0.00E + 00	1.21E−02	ATP6V0D2, ASAH1, CTSA, CTSS, HYAL1, ABCA2, AGA, LAPTM4A, GM2A, DNASE2, CTSH, ARSA, NAPSA, IDS, ATP6V1H, GAA, AP1S3, LAMP3, IDUA, CTSO, AP3B2, LIPA, SUMF1, HGSNAT, NEU1, LAMP2, GUSB, SCARB2, AP1S2, GBA, ACP2, MFSD8, AP4M1, CLN3, LGMN, PSAP
CELL ADHESION MOLECULES CAMS	55	0.444	1.858	0.00E + 00	1.10E−02	ITGB7, NFASC, ITGB8, CDH1, CD86, HLA-C, CLDN4, PVRL2, HLA-B, HLA-F, CD22, CLDN23, OCLN, HLA-A, CNTN1, HLA-E, CLDN18, CLDN1, F11R, CDH3, ITGAV, CLDN16, CD276, PVRL1
INTESTINAL IMMUNE NETWORK FOR IGA PRODUCTION	15	0.573	1.745	1.19E-02	2.99E−02	ITGB7, CD86, TNFSF13B, IL15, IL15RA, CCL28, ICOSLG, HLA-DPA1, CD40

Cluster 3 [ORL-136, ORL-188, ORL-215]

PATHWAY NAME	SIZE	ES	NES	NOM *p*-value	FDR *q*-value	Enriched Genes
RIBOSOME	53	0.582	2.337	0.00E + 00	0.00E + 00	RPS24, RPL37A, RPL9, RPS12, RPL31, RPLP1, RPL34, RPS18, RPL29, RPL12, RPS16, RPL22, RPL10A, RPS27, RPS9, RPL11, RPS21, RPL38, RPS25, RSL24D1, RPL28, RPL14, RPL15, RPS7, RPL37, RPS19, RPL22L1, RPL30, RPL23, RPL18, RPS6, RPL8, RPL13, RPS27A, RPS15
JAK STAT SIGNALING PATHWAY	82	0.403	1.820	0.00E + 00	7.15E−02	IL20, SOCS2, SPRED2, CISH, IL24, IL11, SOCS5, MYC, CTF1, PIAS3, SPRY1, PIM1, IFNGR2, CSF3, PIAS2, CCND2, PIK3CD, SPRED1, IL23A, BCL2L1, SOS2, CCND3, IL3RA
ECM RECEPTOR INTERACTION	46	0.456	1.772	2.25E-03	8.22E−02	FN1, LAMB1, COL4A6, ITGB5, COL6A3, ITGA10, COL1A2, TNC, COL1A1, CD44, COL5A3, SDC4, ITGA7, ITGB1, LAMA3, ITGA2B, COL4A1, LAMC2, ITGA6, HSPG2
FOCAL ADHESION	133	0.361	1.743	0.00E + 00	7.85E−02	DOCK1, GSK3B, FN1, TLN1, PTEN, CAV2, LAMB1, SRC, EGFR, ACTG1, BCL2, ROCK1, COL4A6, RAC3, JUN, ITGB5, PDGFB, COL6A3, CRKL, ITGA10, COL1A2, TNC, CCND2, COL1A1, PIK3CD, FLNB, ELK1, COL5A3, FLNA, VCL, SOS2, ITGA7, PDGFC, CCND3, IGF1R, ITGB1, LAMA3, ZYX, VEGFA, RASGRF1, ITGA2B, COL4A1, LAMC2, MYL2, ITGA6, VEGFB

MAPK SIGNALING PATHWAY	168	0.345	1.711	0.00E + 00	9.12E−02	IL1B, MAPT, PLA2G6, IL1A, CDC25B, MYC, MAP2K5, ARRB2, RASA2, FAS, EGFR, NR4A1, RAC3, JUN, DUSP5, PDGFB, TGFBR1, CRKL, DUSP2, DUSP7, HSPA1L, RRAS, TP53, DUSP16, IKBKG, ATF4, RASGRP2, MAPKAPK2, HSPA2, CACNB3, DUSP6, FLNB, MAPK8IP2, ELK1, MAP3K4, FLNA, TGFBR2, MAP2K7, SOS2, TAOK3, GADD45A, JUND, MAPK7, MAPK8IP1, FGF1, MAP4K4, MAPK11, RPS6KA5, STK4, IKBKB, RASGRF1, TNFRSF1A, RELA, TNF, ELK4, TGFB2, NFATC4, RASA1, NFKB2, TAOK2, RELB, MAPK8IP3, AKT3
MTOR SIGNALING PATHWAY	38	0.407	1.524	1.58E−02	3.30E−01	DDIT4, EIF4B, CAB39, EIF4EBP1, HIF1A, PIK3CD, MLST8, RICTOR, VEGFA, ULK1, VEGFB, TSC2, RPS6, AKT3, PIK3R1
COMPLEMENT AND COAGULATION CASCADES	24	0.444	1.503	4.38E−02	3.26E−01	F2R, THBD, SERPINA1, SERPINF2, SERPING1, F3, C9, CFH, PLAU, CD46, CD55
TYPE I DIABETES MELLITUS	18	0.490	1.502	5.52E−02	2.86E−01	IL1B, IL1A, FAS, PTPRN2, HLA-DPA1, TNF, HLA-E, HLA-DRB1, HLA-DQB1
CYTOSOLIC DNA SENSING PATHWAY	27	0.436	1.499	4.50E−02	2.61E−01	IL1B, POLR3D, POLR3GL, POLR1D, POLR3A, NFKBIA, IKBKG
HYPERTROPHIC CARDIOMYOPATHY HCM	39	0.382	1.454	4.35E−02	3.17E−01	ACTG1, EMD, ITGB5, ITGA10, PRKAG1, CACNB3, TPM4, ITGA7, TPM2, ITGB1, SGCB, TPM1, ITGA2B, TNF, TGFB2, MYL2, ITGA6, PRKAB2, TGFB3, DMD, CACNB1, ITGA2, ACTB

ES - Enrichment Score.
NES - Normalized Enrichment Score.
NOM - Nominal.
FDR - False Discovery Rate.
