# Supplementary material for: Genetically-defined novel oral squamous cell carcinoma cell lines for the development of molecular therapies
Source: Oncotarget. 2016 Apr 1;7(19):27802–18. doi: 10.18632/oncotarget.8533 (PMC5053689; doi:10.18632/oncotarget.8533)
Supplement: Supplementary file 2 [file oncotarget-07-27802-s002.pdf]

Supplementary Table S3: Genomic region of recurrent copy number alterations (CNAs) in ORL lines

Event	Cytoband	Frequency (%)	Affected lines (ORL-)	Overlapping Genes	Reported elsewhere (CNV regions in HNSCC)
Gain	3q23-q24	50.0	215, 153, 174, 188, 150, 195, 156, 196	ATP1B3, TFDP2, GK5, XRN1, ATR, PLS1, TRPC1, PCOLCE2, LOC100507389, PAQR9, LOC100289361, U2SURP, CHST2, SLC9A9, SLC9A9-AS1, C3orf58, PLOD2, PLSCR4, PLSCR2, PLSCR1, PLSCR5	Ambatipudi et al., 2006; Morris et al., 2011, Pickering et al., 2013
Gain	3q24-q25.2	56.3	215, 153, 174, 188, 150, 195, 156, 196, 214	ZIC4, ZIC1, AGTR1, CPB1, CPA3, GYG1, HLTF, HPS3, CP, TM4SF18, TM4SF1, TM4SF4, WWTR1, WWTR1-AS1, COMMD2, ANKUB1, RNF13, PFN2, LOC646903, TSC22D2, SERP1, EIF2A, SELT, FAM194A, SIAH2, CLRN1, CLRN1-AS1, MED12L, GPR171, P2RY14, GPR87, P2RY13, P2RY12, IGSF10, MIR5186, MIR548H2, AADACL2, LOC201651, AADAC, SUCNR1, LOC401093, MBNL1, TMEM14E, P2RY1, RAP2B, C3orf79, ARHGEF26-AS1, ARHGEF26, DHX36, GPR149	Ambatipudi et al., 2006; Morris et al., 2011, Pickering et al., 2013
Gain	3q25.2-q29	62.5	215, 153, 174, 188, 150, 195, 156, 196, 214, 207	MME, LOC100507537, PLCH1, C3orf33, SLC33A1, GMPS, KCNAB1, KCNAB1-AS2, KCNAB1-AS1, SSR3, TIPARP-AS1, TIPARP, LOC730091, PA2G4P4, LEKR1, LOC339894, LOC100498859, CCNL1, VEPH1, PTX3, C3orf55, SHOX2, RSRC1, MLF1, GFM1, LXN, RARRES1, MFSD1, IQCJ, IQCJ-SCHIP1, SCHIP1, MIR3919, IL12A, C3orf80, IFT80, SMC4, MIR15B, MIR16-2, TRIM59, KPNA4, SCARNA7, ARL14, PPM1L, B3GALNT1, NMD3, SPTSSB, OTOL1, LOC647107, SI, SLITRK3, BCHE, ZBBX, SERPINI2, WDR49, PDCD10, SERPINI1, LOC646168, GOLIM4, EGFEM1P, MIR551B, MECOM, TERC, ACTRT3, MYNN, LRRC34, LRRIQ4, LRRC31, SAMD7, LOC100128164, SEC62, GPR160, PHC3, PRKCI, SKIL, CLDN11, SLC7A14, RPL22L1, EIF5A2, SLC2A2, TNIK, MIR569, PLD1, TMEM212, FNDC3B, GHSR, TNFSF10, NCEH1, ECT2, SPATA16, NLGN1, NAALADL2, NAALADL2-AS3, MIR4789, TBL1XR1, LINC00578, KCNMB2-IT1, KCNMB2, ZMAT3, PIK3CA, KCNMB3, ZNF639, MFN1, GNB4, ACTL6A, MRPL47, NDUFB5, USP13, PEX5L, TTC14, CCDC39, FXR1, DNAJC19, SOX2-OT, SOX2, FLJ46066, ATP11B, DCUN1D1, MCCC1, LAMP3, MCF2L2, B3GNT5, LOC100505687, KLHL6, KLHL24, YEATS2, MAP6D1, PARL, ABCC5, ABCC5-AS1, HTR3D, HTR3C, HTR3E, EIF2B5, DVL3, AP2M1, ABCF3, VWA5B2, MIR1224, ALG3, ECE2, CAMK2N2, PSMD2, EIF4G1, SNORD66, FAM131A, CLCN2, POLR2H, THPO, CHRD, EPHB3, MAGEF1, VPS8, C3orf70, EHHADH-AS1, EHHADH, MAP3K13, TMEM41A, LIPH, SENP2, IGF2BP2, C3orf65, TRA2B, LOC344887, ETV5, DGKG, LOC253573, CRYGS, TBCCD1, DNAJB11, AHSG, FETUB, HRG, KNG1, EIF4A2, SNORD2, MIR1248, SNORA81, SNORA63, SNORA4, RFC4, ADIPOQ, ADIPOQ-AS1, ST6GAL1, RPL39L, RTP1, MASP1, RTP4, SST, RTP2, LOC100131635, BCL6, LPP-AS2, LPP, FLJ42393, TPRG1, TPRG1-AS2, TP63, MIR944, LEPREL1, CLDN1, CLDN16, TMEM207, IL1RAP, GMNC, SNAR-I, OSTN, UTS2D, CCDC50, PYDC2, FGF12, MB21D2, HRASLS, MGC2889, ATP13A5, ATP13A4, OPA1, OPA1-AS1, LOC647323, LOC100128023, HES1, LOC100131551, CPN2, LRRC15, GP5, ATP13A3, FLJ34208, LOC100507297, TMEM44, LSG1, FAM43A, LOC100507391, XXYLT1, ACAP2, MIR5692C1, PPP1R2, APOD, SDHAP2, MIR570, MUC20, MUC4, TNK2, SDHAP1, TFRC, LOC401109, ZDHHC19, OSTalpha, PCYT1A, TCTEX1D2, TM4SF19-TCTEX1D2, TM4SF19, UBXN7, RNF168, C3orf43, WDR53, FBXO45, LRRC33, CEP19, PIGX, PAK2, SENP5, NCBP2, LOC152217, PIGZ, MFI2, MFI2-AS1, DLG1, MIR4797, DLG1-AS1, BDH1, LOC220729, KIAA0226, MIR922, FYTTD1, LRCH3, IQCG, RPL35A, LMLN	Ambatipudi et al., 2006; Morris et al., 2011, Pickering et al., 2013
Gain	5p15.33-p13.3	37.5	174, 214, 207, 48, 247, 136	PDCD6, AHRR, C5orf55, EXOC3, PP7080, SLC9A3, MIR4456, CEP72, TPPP, ZDHHC11, BRD9, TRIP13, LOC100506688, NKD2, SLC12A7, MIR4635, SLC6A19, SLC6A18, TERT, MIR4457, CLPTM1L, SLC6A3, LPCAT1, SDHAP3, LOC728613, MIR4277, MRPL36, NDUFS6, IRX4, IRX2, C5orf38, LOC285577, IRX1, LOC340094, ADAMTS16, KIAA0947, FLJ33360, MED10, UBE2QL1, LOC255167, NSUN2, SRD5A1, PAPD7, MIR4278, MIR4454, LOC442132, ADCY2, C5orf49, FASTKD3, MTRR, LOC729506, LOC100505738, MIR4458, SEMA5A, MIR4636, LOC100505806, SNORD123, TAS2R1, LOC285692, FAM173B, CCT5, CMBL, Mar-06, ROPN1L, ANKRD33B, DAP, CTNND2, TAG, DNAH5, TRIO, FAM105A, FAM105B, ANKH, LOC100130744, MIR4637, FBXL7, Mar-11, ZNF622, FAM134B, MYO10, LOC285696, BASP1, LOC401177, CDH18, GUSBP1, CDH12, PMCHL1, PRDM9, CDH10, LOC340107, CDH9, LOC643401, LSP1P3	Ambatipudi et al., 2006; Morris et al., 2011
Gain	5p13.3-p13.1	43.8	174, 214, 207, 48, 247, 136, 195	CDH6, DROSHA, C5orf22, PDZD2, MIR4279, GOLPH3, MTMR12, ZFR, SUB1, NPR3, LOC340113, TARS, ADAMTS12, RXFP3, SLC45A2, AMACR, C1QTNF3-AMACR, C1QTNF3, RAI14, TTC23L, RAD1, BRIX1, DNAJC21, AGXT2, PRLR, SPEF2, IL7R, CAPSL, UGT3A1, UGT3A2, LMBRD2, MIR580, SKP2, NADKD1, RANBP3L, SLC1A3, LOC646719, NIPBL, C5orf42, NUP155, WDR70, GDNF, EGFLAM, EGFLAM-AS4, LIFR, MIR3650, OSMR, RICTOR, FYB, C9, DAB2, PTGER4, TTC33, PRKAA1, LOC100506548, RPL37, SNORD72, CARD6, C7, HEATR7B2, C6	Ambatipudi et al., 2006; Morris et al., 2011
Gain	5p13.1-p11	37.5	174, 214, 207, 48, 247, 136	PLCXD3, OXCT1, C5orf51, FBXO4, GHR, CCDC152, SEPP1, LOC648987, ANXA2R, LOC153684, LOC100132356, ZNF131, NIM1, HMGCS1, CCL28, C5orf28, C5orf34, PAIP1, NNT, FGF10, MRPS30, HCN1	Ambatipudi et al., 2006; Morris et al., 2011
Gain	7p22.3-p22.1	62.5	215, 153, 174, 188, 150, 207, 48, 247, 115, 204	FAM20C, LOC100288524, LOC442497, PDGFA, FLJ44511, PRKAR1B, HEATR2, SUN1, GET4, ADAP1, COX19, CYP2W1, C7orf50, MIR339, GPR146, GPER, ZFAND2A, UNCX, MICALL2, INTS1, MAFK, TMEM184A, PSMG3, KIAA1908, TFAMP1, ELFN1, MAD1L1, MIR4655, FTSJ2, NUDT1, SNX8, EIF3B, CHST12, LFNG, MIR4648, BRAT1, IQCE, TTYH3, AMZ1, GNA12, CARD11, SDK1, FOXK1, AP5Z1, MIR4656, RADIL, PAPOLB, MMD2, RNF216P1, RBAK, RBAK-LOC389458, LOC389458, ZNF890P, WIPI2, SLC29A4, TNRC18, FBXL18, MIR589, ACTB, FSCN1, RNF216, RNF216-IT1, ZNF815P, OCM, CCZ1, RSPH10B, RSPH10B2, PMS2, AIMP2, EIF2AK1, USP42, CYTH3, FAM220A, RAC1, DAGLB, KDELR2, GRID2IP, ZDHHC4, C7orf26, ZNF853, ZNF12, PMS2CL, CCZ1B, LOC100131257, C1GALT1	Ambatipudi et al., 2006; Morris et al., 2011
Gain	7p21.3-p11.2	56.3	215, 153, 174, 188, 207, 48, 247, 115, 204<br>215, 153, 174, 188, 150, 195, 156, 214, 207, 48, 247, 115, 136, 204, 166	PER4, NDUFA4, PHF14, THSD7A, TMEM106B, VWDE, SCIN, ARL4A, ETV1, DGKB, AGMO, MEOX2, ISPD, LOC100506025, SOSTDC1, LRRC72, ANKMY2, BZW2, TSPAN13, AGR2, AGR3, AHR, SNX13, PRPS1L1, HDAC9, TWIST1, FERD3L, TWISTNB, MIR3146, TMEM196, MACC1, MACC1-AS1, ITGB8, ABCB5, SP8, RPL23P8, SP4, DNAH11, CDCA7L, RAPGEF5, STEAP1B, LOC100506178, IL6, TOMM7, SNORD93, FAM126A, KLHL7-AS1, KLHL7, NUPL2, GPNMB, MALSU1, IGF2BP3, RPS2P32, TRA2A, CLK2P, CCDC126, FAM221A, STK31, NPY, MPP6, DFNA5, OSBPL3, CYCS, C7orf31, NPVF, RNU6-16, MIR148A, NFE2L3, HNRNPA2B1, CBX3, SNX10, LOC441204, KIAA0087, C7orf71, SKAP2, HOXA1, HOTAIRM1, HOXA2, HOXA3, HOXA4, HOXA-AS3, HOXA5, HOXA6, HOXA7, HOXA9, HOXA10-HOXA9, HOXA-AS4, MIR196B, HOXA10, HOXA11, HOXA11-AS, HOXA13, HOTTIP, EVX1, HIBADH, TAX1BP1, JAZF1, JAZF1-AS1, CREB5, TRIL, LOC100506497, CPVL, CHN2, PRR15, LOC646762, MIR550A3, ZNRF2P2, DPY19L2P3, WIPF3, SCRN1, FKBP14, PLEKHA8, C7orf41, ZNRF2, MIR550A1, MIR550B1, DKFZP586I1420, NOD1, GGCT, LOC401320, GARS, CRHR2, INMT, INMT-FAM188B, FAM188B, AQP1, GHRHR, ADCYAP1R1, NEUROD6, CCDC129, PPP1R17, PDE1C, LOC100130673, LSM5, AVL9, DPY19L1P1, ZNRF2P1, MIR550B2, MIR550A2, LOC401321, KBTBD2, RP9P, FKBP9, NT5C3, RP9, BBS9, BMPER, NPSR1-AS1, NPSR1, DPY19L1, DPY19L2P1, TBX20, LOC401324, HERPUD2, Sep-07, EEPD1, KIAA0895, ANLN, AOAH, AOAH-IT1, ELMO1, MIR1200, GPR141, NME8, SFRP4, EPDR1, STARD3NL, TARP, LOC100506776, AMPH, FAM183B, VPS41, POU6F2, POU6F2-AS1, YAE1D1, LOC646999, RALA, LINC00265, CDK13, MPLKIP, C7orf10, INHBA, INHBA-AS1, GLI3, C7orf25, PSMA2, MRPL32, HECW1, MIR3943, LOC100506895, STK17A, COA1, BLVRA, MRPS24, URGCP-MRPS24, URGCP, UBE2D4, POLR2J4, SPDYE1, RASA4P, FLJ35390, DBNL, PGAM2, POLM, AEBP1, MIR4649, POLD2, MYL7, GCK, YKT6, CAMK2B, NUDCD3, NPC1L1, DDX56, TMED4, OGDH, ZMIZ2, PPIA, H2AFV, PURB, MIR4657, MYO1G, SNHG15, SNORA9, CCM2, NACAD, TBRG4, SNORA5A, SNORA5C, SNORA5B, RAMP3, ADCY1, SEPT7P2, IGFBP1, IGFBP3, TNS3, C7orf65, LINC00525, PKD1L1, C7orf69, HUS1, SUN3, C7orf57, UPP1, ABCA13, CDC14C, VWC2, ZPBP, C7orf72, IKZF1, FIGNL1, DDC, LOC100129427, GRB10, COBL, POM121L12, FLJ45974, HPVC1, VSTM2A, LOC285878, SEC61G, EGFR, LOC100507500, LANCL2, VOPP1, FKBP9L, Sep-14, ZNF713, MRPS17, GBAS, PSPH, CCT6A, SNORA15, SUMF2, PHKG1, CHCHD2, LOC389493, LOC650226, DKFZp434L192, LOC100130849, MIR4283-2, MIR4283-1, ZNF479, GUSBP10, MIR3147, ZNF716	Ambatipudi et al., 2006; Morris et al., 2011, Pickering et al., 2013
Gain	8q11.21-q23.3	93.8		KIAA0146, CEBPD, PRKDC, MCM4, UBE2V2, EFCAB1, SNAI2, C8orf22, SNTG1, PXDNL, PCMTD1, ST18, FAM150A, RB1CC1, NPBWR1, OPRK1, ATP6V1H, RGS20, TCEA1, LYPLA1, MRPL15, SOX17, RP1, XKR4, SBF1P1, TMEM68, TGS1, LYN, RPS20, SNORD54, MOS, PLAG1, CHCHD7, SDR16C5, PENK, LOC100507632, IMPAD1, LOC100507651, LOC286177, LINC00588, FAM110B, UBXN2B, CYP7A1, SDCBP, NSMAF, TOX, CA8, RAB2A, CHD7, LOC100130298, CLVS1, ASPH, MIR4470, NKAIN3, UG0898H09, GGH, TTPA, YTHDF3, LOC286184, LOC100130155, MIR124-2, LOC401463, BHLHE22, CYP7B1, LINC00251, LOC286186, ARMC1, MTFR1, PDE7A, DNAJC5B, TRIM55, CRH, LOC100505659, LOC100505676, RRS1, ADHFE1, C8orf46, MYBL1, VCPIP1, C8orf44, C8orf44-SGK3, SGK3, PTTG3P, MCMDC2, SNHG6, SNORD87, TCF24, PPP1R42, COPS5, CSPP1, ARFGEF1, CPA6, PREX2, LOC286189, C8orf34, LOC100505718, SULF1, SLCO5A1, PRDM14, NCOA2, TRAM1, LOC286190, LACTB2, XKR9, EYA1, MSC, LOC100132891, RNU6-83, TRPA1, LOC392232, KCNB2, TERF1, SBSPON, LOC100130301, RPL7, RDH10, STAU2-AS1, STAU2, UBE2W, TCEB1, TMEM70, LY96, JPH1, GDAP1, MIR5681A, MIR5681B, FLJ39080, MIR2052, PI15, CRISPLD1, HNF4G, LOC100192378, ZFHX4, PEX2, PKIA, ZC2HC1A, IL7, STMN2, HEY1, MRPS28, TPD52, MIR5708, ZBTB10, ZNF704, PAG1, FABP5, PMP2, FABP9, FABP4, FABP12, IMPA1, SLC10A5, ZFAND1, CHMP4C, SNX16, RALYL, LRRCC1, E2F5, C8orf59, CA13, CA1, CA3, CA2, REXO1L2P, REXO1L1, PSKH2, ATP6V0D2, SLC7A13, WWP1, FAM82B, CPNE3, CNGB3, CNBD1, DCAF4L2, MMP16, RIPK2, OSGIN2, NBN, DECR1, CALB1, LINC00534, TMEM64, NECAB1, LOC100127983, TMEM55A, OTUD6B, LRRC69, MIR4661, SLC26A7, RUNX1T1, FLJ46284, TRIQK, C8orf87, LINC00535, FAM92A1, RBM12B, RBM12B-AS1, TMEM67, MIR378D2, PDP1, CDH17, GEM, RAD54B, FSBP, KIAA1429, LOC100288748, ESRP1, DPY19L4, INTS8, CCNE2, TP53INP1, NDUFAF6, MIR3150B, MIR3150A, PLEKHF2, C8orf69, C8orf37, LOC100616530, LOC100500773, GDF6, UQCRB, MTERFD1, PTDSS1, SDC2, CPQ, TSPYL5, MTDH, LAPTM4B, MATN2, RPL30, SNORA72, C8orf47, HRSP12, POP1, NIPAL2, KCNS2, STK3, OSR2, VPS13B, MIR599, MIR875, COX6C, RGS22, FBXO43, POLR2K, SPAG1, RNF19A, MIR4471, ANKRD46, SNX31, PABPC1, YWHAZ, FLJ42969, ZNF706, NACAP1, GRHL2, NCALD, MIR5680, RRM2B, UBR5, ODF1, KLF10, AZIN1, ATP6V1C1, C8orf56, BAALC, MIR3151, LOC100499183, FZD6, CTHRC1, SLC25A32, DCAF13, RIMS2, DCSTAMP, DPYS, LRP12, ZFPM2, OXR1, ABRA, ANGPT1, RSPO2, EIF3E, EMC2, TMEM74, TRHR, NUDCD1, ENY2, PKHD1L1, EBAG9, SYBU, KCNV1, CSMD3, MIR2053	Ambatipudi et al., 2006; Morris et al., 2011, Pickering et al., 2013
Gain	8q23.3-q24.3	100.0	215, 153, 174, 188, 150, 195, 156, 196, 214, 207, 48, 247, 115, 136, 204, 166	TRPS1, LINC00536, EIF3H, UTP23, RAD21, RAD21-AS1, MIR3610, AARD, SLC30A8, MED30, EXT1, SAMD12, SAMD12-AS1, TNFRSF11B, COLEC10, MAL2, NOV, ENPP2, TAF2, DSCC1, DEPTOR, COL14A1, MRPL13, MTBP, SNTB1, HAS2, HAS2-AS1, ZHX2, DERL1, WDR67, FAM83A, LOC100131726, MIR4663, C8orf76, ZHX1-C8ORF76, ZHX1, ATAD2, WDYHV1, FBXO32, KLHL38, ANXA13, FAM91A1, FER1L6, FER1L6-AS1, TMEM65, TRMT12, RNF139, TATDN1, NDUFB9, MTSS1, LOC157381, ZNF572, SQLE, KIAA0196, NSMCE2, TRIB1, LOC100130231, FAM84B, PCAT1, POU5F1B, LOC727677, MYC, MIR1204, PVT1, MIR1205, MIR1206, MIR1207, MIR1208, LOC728724, GSDMC, FAM49B, MIR5194, ASAP1, LOC100507117, ASAP1-IT1, ADCY8, EFR3A, OC90, HHLA1, KCNQ3, HPYR1, LRRC6, TMEM71, PHF20L1, TG, SLA, WISP1, NDRG1, ST3GAL1, ZFAT, ZFAT-AS1, MIR30B, MIR30D, LOC286094, KHDRBS3, FAM135B, COL22A1, KCNK9, TRAPPC9, CHRAC1, EIF2C2, PTK2, DENND3, SLC45A4, LOC731779, GPR20, PTP4A3, FLJ43860, MIR4472-1, LINC00051, TSNARE1, BAI1, ARC, JRK, PSCA, LY6K, LOC100288181, THEM6, SLURP1, LYPD2, LYNX1, LY6D, GML, CYP11B1, CYP11B2, LOC100133669, LY6E, C8orf31, LY6H, GPIHBP1, ZFP41, GLI4, ZNF696, TOP1MT, RHPN1-AS1, RHPN1, MAFA, ZC3H3, GSDMD, C8orf73, NAPRT1, EEF1D, TIGD5, PYCRL, TSTA3, ZNF623, ZNF707, BREA2, CCDC166, MAPK15, FAM83H, MIR4664, LOC100128338, SCRIB, MIR937, PUF60, NRBP2, EPPK1, PLEC, MIR661, PARP10, GRINA, SPATC1, OPLAH, EXOSC4, GPAA1, CYC1, SHARPIN, MAF1, KIAA1875, FAM203A, HEATR7A, SCXB, SCXA, BOP1, HSF1, DGAT1, SCRT1, C8ORFK29, TMEM249, FBXL6, SLC52A2, ADCK5, CPSF1, MIR939, MIR1234, SLC39A4, VPS28, TONSL, CYHR1, KIFC2, FOXH1, PPP1R16A, GPT, MFSD3, RECQL4, LRRC14, LRRC24, C8orf82, ARHGAP39, ZNF251, ZNF34, RPL8, ZNF517, ZNF7, COMMD5, ZNF250, ZNF16, ZNF252P, TMED10P1, ZNF252P-AS1, C8orf33	Ambatipudi et al., 2006; Morris et al., 2011, Pickering et al., 2013
Gain	9p13.1-p11.2	37.5	215, 153, 156, 196, 48, 247	CNTNAP3, FAM75A1, FAM75A2, LOC653501, ZNF658B, FAM74A1, FAM75A3, FAM74A3, ZNF658, FAM75A5, FAM75A4, FAM75A7, MGC21881, KGFLP2, LOC643648, ANKRD20A2, ANKRD20A3, FAM95B1, FOXD4L4, FOXD4L2, LOC286297, AQP7P3, LOC642929, FAM75A6, CNTNAP3B, FAM27C, FAM27A, KGFLP1, LOC643648	
Gain	9q12-q13	37.5	215, 153, 156, 196, 48, 247	FAM74A2, FAM74A4, FAM75A5, FAM75A7, PTGER4P2, LOC286297, AQP7P1, FAM27B, ANKRD20A1, ANKRD20A3, LOC642236	Ambatipudi et al., 2006, Pickering et al., 2013
Gain	9q13-q21.33	43.8	215, 153, 156, 196, 48, 247, 214	LOC100132352, PGM5P2, LOC440896, FOXD4L6, CBWD6, ANKRD20A4, LOC100133920, FOXD4L5, FOXD4L4, FOXD4L2, CBWD3, CBWD5, FOXD4L3, LOC572558, PGM5, C9orf71, PIP5K1B, FAM122A, PRKACG, FXN, TJP2, BANCR, FAM189A2, APBA1, PTAR1, LOC494558, C9orf135, MAMDC2, LOC100507244, LOC100507299, SMC5, KLF9, TRPM3, MIR204, TMEM2, FAM108B1, C9orf85, C9orf57, GDA, ZFAND5, TMC1, ALDH1A1, ANXA1, RORB, TRPM6, C9orf40, C9orf41, NMRK1, OSTF1, MIR548H3, PCSK5, RFK, RPSAP9, GCNT1, PRUNE2, PCA3, FOXB2, LOC100286938, VPS13A, GNA14, GNAQ, CEP78, PSAT1, TLE4, TLE1, FAM75D5, FAM75D4, FAM75D3, FAM75D1, RASEF, FRMD3, IDNK, UBQLN1, GKAP1, KIF27, C9orf64, HNRNPK, MIR7-1, RMI1, SLC28A3, NTRK2	Ambatipudi et al., 2006, Pickering et al., 2013
Gain	9q21.33-q34.3	56.3	215, 153, 174, 195, 156, 196, 48, 247, 214	AGTPBP1, LOC389765, NAA35, GOLM1, C9orf153, ISCA1, ZCCHC6, GAS1, LOC100506834, LOC440173, LOC494127, C9orf170, DAPK1, CTSL1, CTSL3, CTSL1P8, LOC392364, FAM75E1, FAM75C1, CDK20, FAM75C2, SPIN1, NXNL2, LOC286238, MIR4289, C9orf47, S1PR3, SHC3, CKS2, MIR3153, SECISBP2, SEMA4D, GADD45G, UNQ6494, LOC286370, MIR4290, LOC340515, DIRAS2, SYK, LOC100129316, AUH, NFIL3, MIR3910-1, MIR3910-2, ROR2, SPTLC1, LOC100128076, LINC00475, IARS, MIR3651, SNORA84, NOL8, CENPP, OGN, OMD, ASPN, ECM2, MIR4670, IPPK, LOC100128361, BICD2, ANKRD19P, ZNF484, FGD3, SUSD3, C9orf89, NINJ1, WNK2, C9orf129, FAM120AOS, FAM120A, PHF2, MIR4291, BARX1, PTPDC1, MIRLET7A1, MIRLET7F1, MIRLET7DHG, MIRLET7D, ZNF169, FAM22F, LOC100132077, HIATL1, FBP2, FBP1, C9orf3, MIR2278, MIR23B, MIR27B, MIR3074, MIR24-1, FANCC, PTCH1, LOC100507346, LINC00476, ERCC6L2, LINC00092, LOC158435, LOC158434, HSD17B3, SLC35D2, ZNF367, HABP4, CDC14B, AAED1, LOC441455, ZNF510, ZNF782, LOC100132781, LOC441454, FAM22G, HIATL2, CTSL2, LOC340508, LOC100499484, LOC100499484-C9ORF174, C9orf174, LOC286359, TDRD7, TMOD1, TSTD2, NCBP1, XPA, FOXE1, C9orf156, HEMGN, ANP32B, NANS, TRIM14, CORO2A, TBC1D2, GABBR2, ANKS6, GALNT12, COL15A1, TGFBR1, ALG2, SEC61B, NR4A3, LOC441461, STX17, ERP44, INVS, TEX10, MSANTD3, MSANTD3-TMEFF1, TMEFF1, MURC, LPPR1, BAAT, MRPL50, ZNF189, ALDOB, TMEM246, RNF20, GRIN3A, PPP3R2, CYLC2, SMC2, OR13F1, OR13C4, OR13C3, OR13C8, OR13C5, OR13C2, OR13C9, OR13D1, NIPSNAP3A, NIPSNAP3B, LOC286367, ABCA1, SLC44A1, FSD1L, FKTN, TAL2, TMEM38B, ZNF462, MIR548Q, RAD23B, KLF4, ACTL7B, ACTL7A, IKBKAP, FAM206A, CTNNAL1, TMEM245, MIR32, FRRS1L, EPB41L4B, PTPN3, PALM2, PALM2-AKAP2, AKAP2, C9orf152, TXN, TXNDC8, SVEP1, MUSK, LPAR1, OR2K2, KIAA0368, ZNF483, PTGR1, LRRC37A5P, DNAJC25, DNAJC25-GNG10, GNG10, C9orf84, UGCG, MIR4668, MIR3134, SUSD1, PTBP3, HSDL2, KIAA1958, INIP, SNX30, SLC46A2, ZNF883, ZFP37, FAM225B, LINC00256A, SLC31A2, FKBP15, SLC31A1, CDC26, PRPF4, RNF183, WDR31, BSPRY, HDHD3, ALAD, POLE3, C9orf43, RGS3, ZNF618, AMBP, KIF12, COL27A1, MIR455, ORM1, ORM2, AKNA, DFNB31, ATP6V1G1, C9orf91, LOC100505478, TNFSF15, TNFSF8, TNC, Dec-01, LINC00474, PAPPA, ASTN2, LOC100128505, TRIM32, SNORA70C, TLR4, DBC1, MIR147A, CDK5RAP2, MEGF9, FBXW2, LOC100288842, PSMD5, LOC253039, PHF19, TRAF1, C5, CNTRL, RAB14, GSN, STOM, GGTA1P, DAB2IP, TTLL11, MIR4478, NDUFA8, MORN5, LHX6, RBM18, MRRF, PTGS1, OR1J1, OR1J2, OR1J4, OR1N1, OR1N2, OR1L8, OR1Q1, OR1B1, OR1L1, OR1L3, OR1L4, OR1L6, OR5C1, OR1K1, PDCL, RC3H2, SNORD90, ZBTB6, ZBTB26, RABGAP1, GPR21, MIR600HG, MIR600, STRBP, CRB2, DENND1A, MIR601, LHX2, NEK6, PSMB7, LOC100129034, GPR144, NR5A1, NR6A1, MIR181A2HG, MIR181A2, MIR181B2, OLFML2A, WDR38, RPL35, ARPC5L, GOLGA1, SCAI, PPP6C, RABEPK, HSPA5, GAPVD1, MAPKAP1, PBX3, FAM125B, NRON, LMX1B, ZBTB43, ZBTB34, RALGPS1, ANGPTL2, GARNL3, SLC2A8, ZNF79, RPL12, SNORA65, LRSAM1, FAM129B, STXBP1, MIR3911, C9orf117, PTRH1, TTC16, TOR2A, SH2D3C, MIR3960, MIR2861, CDK9, FPGS, ENG, AK1, MIR4672, ST6GALNAC6, ST6GALNAC4, PIP5KL1, DPM2, FAM102A, NAIF1, SLC25A25, LOC100289019, PTGES2, LOC389791, LCN2, C9orf16, CIZ1, DNM1, MIR199B, MIR3154, GOLGA2, SWI5, TRUB2, COQ4, SLC27A4, URM1, MIR219-2, MIR2964A, CERCAM, ODF2, GLE1, SPTAN1, WDR34, SET, PKN3, ZDHHC12, LOC100506100, ZER1, TBC1D13, ENDOG, C9orf114, CCBL1, LRRC8A, PHYHD1, DOLK, NUP188, SH3GLB2, FAM73B, DOLPP1, CRAT, PPP2R4, IER5L, C9orf106, LOC100506190, C9orf50, NTMT1, ASB6, PRRX2, PTGES, TOR1B, TOR1A, C9orf78, USP20, FNBP1, GPR107, NCS1, ASS1, LOC100272217, FUBP3, PRDM12, EXOSC2, ABL1, QRFP, FIBCD1, LAMC3, AIF1L, NUP214, FAM78A, PPAPDC3, PRRC2B, SNORD62A, SNORD62B, POMT1, UCK1, RAPGEF1, MED27, NTNG2, SETX, TTF1, C9orf171, BARHL1, DDX31, GTF3C4, AK8, C9orf9, TSC1, GFI1B, GTF3C5, CEL, CELP, RALGDS, GBGT1, OBP2B, ABO, SURF6, MED22, RPL7A, SNORD24, SNORD36B, SNORD36A, SNORD36C, SURF1, SURF2, SURF4, C9orf96, REXO4, ADAMTS13, CACFD1, SLC2A6, TMEM8C, ADAMTSL2, FAM163B, DBH, SARDH, VAV2, LINC00094, BRD3, WDR5, RNU6ATAC, RXRA, MIR4669, COL5A1, MIR3689A, MIR3689C, MIR3689D1, MIR3689B, MIR3689D2, MIR3689E, MIR3689F, FCN2, FCN1, OLFM1, LOC401557, C9orf62, LOC100506599, PPP1R26, C9orf116, MRPS2, LCN1, OBP2A, PAEP, LOC100130954, GLT6D1, LCN9, SOHLH1, KCNT1, CAMSAP1, UBAC1, NACC2, C9orf69, LHX3, QSOX2, DKFZP434A062, GPSM1, DNLZ, CARD9, SNAPC4, SDCCAG3, PMPCA, INPP5E, SEC16A, C9orf163, NOTCH1, MIR4673, MIR4674, EGFL7, MIR126, AGPAT2, FAM69B, SNHG7, SNORA43, SNORA17, LCN10, LCN6, LOC100128593, LCN8, LCN15, TMEM141, KIAA1984, KIAA1984-AS1, C9orf86, MIR4292, C9orf172, PHPT1, MAMDC4, EDF1, TRAF2, MIR4479, FBXW5, C8G, LCN12, PTGDS, LCNL1, C9orf142, CLIC3, ABCA2, C9orf139, FUT7, NPDC1, ENTPD2, SAPCD2, UAP1L1, LOC100289341, MAN1B1, DPP7, GRIN1, LRRC26, MIR3621, ANAPC2, SSNA1, TPRN, TMEM203, NDOR1, RNF208, C9orf169, RNF224, SLC34A3, TUBB4B, FAM166A, LOC100129722, C9orf173, COBRA1, TOR4A, NRARP, EXD3, NOXA1, ENTPD8, NELF, PNPLA7, MRPL41, WDR85, ZMYND19, ARRDC1, C9orf37, EHMT1, FLJ40292, MIR602, CACNA1B, TUBBP5	Ambatipudi et al., 2006, Pickering et al., 2013
Gain	11q12.2-q13.1	37.5	174, 195, 207, 48, 136, 166	RAB3IL1, BEST1, FTH1, INCENP, SCGB1D1, SCGB2A1, SCGB1D2, SCGB2A2, SCGB1D4, ASRGL1, SCGB1A1, AHNAK, EEF1G, MIR3654, TUT1, MTA2, EML3, ROM1, B3GAT3, GANAB, INTS5, C11orf48, METTL12, SNORA57, C11orf83, UBXN1, LRRN4CL, BSCL2, HNRNPUL2-BSCL2, GNG3, HNRNPUL2, TTC9C, ZBTB3, POLR2G, TAF6L, TMEM179B, TMEM223, NXF1, STX5, WDR74, SNHG1, SNORD22, SNORD31, SNORD30, SNORD29, SNORD28, SNORD27, SNORD26, SNORD25, SLC3A2, CHRM1, SLC22A6, SLC22A8, SLC22A24, SLC22A25, MIR3680-2, MIR3680-1, SLC22A10, SLC22A9, HRASLS5, LGALS12, RARRES3, HRASLS2, PLA2G16, ATL3, RTN3, C11orf95, C11orf84, MARK2, RCOR2, NAA40, COX8A, OTUB1, MACROD1, FLRT1, STIP1, FERMT3, TRPT1, NUDT22, DNAJC4, VEGFB, FKBP2, PPP1R14B, PLCB3, BAD, GPR137, KCNK4, C11orf20, ESRRA, TRMT112, PRDX5, CCDC88B	Ambatipudi et al., 2006; Morris et al., 2011
Gain	11q13.1-q13.2	50.0	174, 188, 195, 207, 48, 136, 204, 166	RPS6KA4, MIR1237, SLC22A11, SLC22A12, NRXN2, RASGRP2, PYGM, SF1, MAP4K2, MEN1, CDC42BPG, EHD1, MIR192, MIR194-2, ATG2A, PPP2R5B, GPHA2, C11orf85, BATF2, ARL2, ARL2-SNX15, SNX15, SAC3D1, NAALADL1, CDCA5, ZFPL1, LOC100130348, C11orf2, TM7SF2, ZNHIT2, FAU, MRPL49, SYVN1, SPDYC, CAPN1, SLC22A20, POLA2, CDC42EP2, DPF2, TIGD3, SLC25A45, FRMD8, NEAT1, MIR612, MALAT1, MIR548AR, SCYL1, LTBP3, LOC254100, SSSCA1, FAM89B, EHBP1L1, KCNK7, MAP3K11, PCNXL3, MIR4690, SIPA1, MIR4489, RELA, KAT5, RNASEH2C, AP5B1, OVOL1, SNX32, CFL1, MUS81, EFEMP2, CTSW, FIBP, CCDC85B, FOSL1, C11orf68, DRAP1, TSGA10IP, SART1, EIF1AD, BANF1, CST6, CATSPER1, GAL3ST3, SF3B2, PACS1, KLC2, RAB1B, CNIH2, YIF1A, TMEM151A, CD248, RIN1, BRMS1, B3GNT1, SLC29A2, NPAS4, MRPL11, PELI3, DPP3, BBS1, ZDHHC24, ACTN3, CTSF, CCDC87, CCS, RBM14, RBM14-RBM4, RBM4, RBM4B, SPTBN2, C11orf80, RCE1, PC, LRFN4, C11orf86, SYT12, RHOD, KDM2A, ADRBK1, ANKRD13D, SSH3, LOC100130987, POLD4, CLCF1, RAD9A, PPP1CA, TBC1D10C, CARNS1, RPS6KB2, PTPRCAP, CORO1B, GPR152, CABP4, TMEM134, AIP, PITPNM1, CDK2AP2, CABP2, GSTP1, NDUFV1, DOC2GP, NUDT8, TBX10, ACY3, ALDH3B2, FAM86C2P, UNC93B1, ALDH3B1, NDUFS8, MIR4691, TCIRG1, CHKA, SUV420H1, C11orf24, LRP5, PPP6R3	Ambatipudi et al., 2006; Morris et al., 2011, Pickering et al., 2013
Gain	11q13.2-q13.4	62.5	153, 174, 188, 195, 207, 48, 247, 136, 204, 166	GAL, MTL5, CPT1A, MRPL21, IGHMBP2, MRGPRD, MRGPRF, TPCN2, MYEOV, CCND1, ORAOV1, FGF19, FGF4, FGF3, ANO1, FADD, PPFIA1, MIR548K, CTTN, SHANK2, SHANK2-AS1, MIR3664	Ambatipudi et al., 2006; Morris et al., 2011, Pickering et al., 2013
Gain	17q21.31	50.0	153, 150, 195, 156, 214, 48, 115, 136	KANSL1, KANSL1-AS1, LRRC37A, ARL17A, ARL17B, NSFP1, LRRC37A2, NSF	
Gain	20p13-p11.1	68.8	215, 153, 174, 188, 196, 207, 247, 115, 136, 204, 166	DEFB128, DEFB129, DEFB132, C20orf96, ZCCHC3, SOX12, NRSN2, TRIB3, RBCK1, TBC1D20, CSNK2A1, TCF15, SRXN1, SCRT2, SLC52A3, FAM110A, ANGPT4, RSPO4, PSMF1, TMEM74B, C20orf202, RAD21L1, SNPH, SDCBP2, FKBP1A-SDCBP2, LOC100507495, FKBP1A, NSFL1C, SIRPB2, SIRPD, SIRPB1, SIRPG, LOC100289473, SIRPA, PDYN, STK35, TGM3, TGM6, SNRPB, SNORD119, ZNF343, TMC2, NOP56, MIR1292, SNORD110, SNORA51, SNORD86, SNORD56, SNORD57, IDH3B, EBF4, CPXM1, C20orf141, TMEM239, PCED1A, VPS16, PTPRA, GNRH2, MRPS26, OXT, AVP, UBOX5-AS1, UBOX5, FASTKD5, PROSAPIP1, DDRGK1, ITPA, SLC4A11, C20orf194, ATRN, GFRA4, ADAM33, SIGLEC1, HSPA12B, C20orf27, SPEF1, CENPB, CDC25B, AP5S1, MAVS, PANK2, MIR103A2, MIR103B2, RNF24, SMOX, LOC728228, ADRA1D, PRNP, PRND, PRNT, RASSF2, SLC23A2, TMEM230, PCNA, PCNA-AS1, CDS2, PROKR2, LOC100507629, LOC643406, LOC149837, GPCPD1, C20orf196, CHGB, TRMT6, MCM8, CRLS1, LRRN4, FERMT1, BMP2, HAO1, TMX4, PLCB1, PLCB4, LAMP5, PAK7, LOC100131208, ANKRD5, SNAP25, MKKS, C20orf94, JAG1, LOC339593, BTBD3, SPTLC3, ISM1, LOC100505536, TASP1, ESF1, NDUFAF5, SEL1L2, MACROD2, FLRT3, MACROD2-AS1, KIF16B, SNRPB2, OTOR, PCSK2, BFSP1, DSTN, RRBP1, BANF2, SNX5, SNORD17, C20orf72, OVOL2, PET117, CSRP2BP, ZNF133, DZANK1-AS1, DZANK1, POLR3F, MIR3192, RBBP9, SEC23B, LINC00493, DTD1, HSPC072, LOC100270804, C20orf78, C20orf79, SLC24A3, LOC100130264, RIN2, NAA20, CRNKL1, C20orf26, INSM1, RALGAPA2, PLK1S1, XRN2, NKX2-4, NKX2-2, PAX1, LOC100270679, LOC284788, LINC00261, FOXA2, SSTR4, THBD, CD93, LOC200261, NXT1, GZF1, NAPB, CSTL1, CST11, CST8, CST7, CST9L, CST9, CST3, CST4, CST1, CST2, CST5, GGTLC1, FLJ33581, SYNDIG1, CST7, C20orf3, ACSS1, VSX1, LOC284798, ENTPD6, PYGB, ABHD12, GINS1, NINL, NANP, ZNF337, FAM182B, LOC100134868, FAM182A, NCOR1P1, LOC284801, MIR663A	Ambatipudi et al., 2006; Morris et al., 2011, Pickering et al., 2013
Gain	20q11.21-q11.23	81.3	215, 153, 174, 188, 195, 196, 207, 48, 247, 115, 136, 204, 166	FRG1B, MLLT10P1, DEFB115, DEFB116, DEFB118, DEFB119, DEFB121, DEFB122, DEFB123, DEFB124, REM1, LINC00028, HM13, PSIMCT-1, HM13-AS1, ID1, MIR3193, COX4I2, BCL2L1, TPX2, MYLK2, FOXS1, DUSP15, TTLL9, PDRG1, XKR7, C20orf160, HCK, TM9SF4, TSPY26P, PLAGL2, POFUT1, KIF3B, ASXL1, C20orf112, LOC149950, C20orf203, COMMD7, DNMT3B, MAPRE1, SUN5, BPIFB2, BPIFB6, BPIFB3, BPIFB4, BPIFA2, BPIFA4P, BPIFA3, BPIFA1, BPIFB1, CDK5RAP1, SNTA1, CBFA2T2, NECAB3, C20orf144, ACTL10, E2F1, PXMP4, ZNF341, CHMP4B, RALY, MIR4755, EIF2S2, ASIP, AHCY, ITCH, MIR644A, DYNLRB1, MAP1LC3A, PIGU, TP53INP2, NCOA6, HMGB3P1, GGT7, ACSS2, GSS, MYH7B, MIR499A, MIR499B, TRPC4AP, EDEM2, PROCR, MMP24, EIF6, FAM83C, UQCC, GDF5, CEP250, C20orf173, ERGIC3, FER1L4, SPAG4, CPNE1, RBM12, NFS1, ROMO1, RBM39, PHF20, SCAND1, C20orf152, LOC647979, EPB41L1, C20orf4, DLGAP4, MYL9, TGIF2, TGIF2-C20ORF24, C20orf24, SLA2, NDRG3, DSN1, SOGA1	Ambatipudi et al., 2006; Morris et al., 2011, Pickering et al., 2013
Gain	20q11.23-q13.33	75.0	215, 153, 174, 188, 195, 196, 48, 247, 115, 136, 204, 166	C20orf118, SAMHD1, RBL1, C20orf132, RPN2, GHRH, MANBAL, SRC, BLCAP, NNAT, LINC00489, LOC100287792, CTNNBL1, VSTM2L, TTI1, RPRD1B, TGM2, KIAA1755, BPI, LBP, LOC388796, SNORA71B, SNORA71A, SNORA71C, SNORA71D, SNHG11, SNORA39, SNORA60, RALGAPB, ADIG, ARHGAP40, SLC32A1, ACTR5, PPP1R16B, FAM83D, DHX35, LOC339568, MAFB, TOP1, PLCG1, ZHX3, LPIN3, EMILIN3, CHD6, PTPRT, SRSF6, L3MBTL1, SGK2, IFT52, MYBL2, GTSF1L, TOX2, JPH2, C20orf111, LOC100505783, GDAP1L1, FITM2, R3HDML, HNF4A, MIR3646, TTPAL, SERINC3, PKIG, ADA, LOC79015, WISP2, KCNK15, RIMS4, YWHAB, PABPC1L, TOMM34, STK4-AS1, STK4, KCNS1, WFDC5, WFDC12, PI3, SEMG1, SEMG2, SLPI, MATN4, RBPJL, SDC4, SYS1, SYS1-DBNDD2, TP53TG5, DBNDD2, PIGT, WFDC2, SPINT3, WFDC6, SPINLW1-WFDC6, SPINLW1, WFDC8, WFDC9, WFDC10A, WFDC11, WFDC10B, WFDC13, SPINT4, WFDC3, DNTTIP1, UBE2C, TNNC2, SNX21, ACOT8, ZSWIM3, ZSWIM1, SPATA25, NEURL2, CTSA, PLTP, PCIF1, ZNF335, MMP9, SLC12A5, NCOA5, CD40, CDH22, SLC35C2, ELMO2, ZNF663, MKRN7P, ZNF334, OCSTAMP, SLC13A3, TP53RK, SLC2A10, EYA2, MIR3616, ZMYND8, LOC100131496, NCOA3, SULF2, LINC00494, PREX1, ARFGEF2, CSE1L, STAU1, DDX27, ZNFX1, ZNFX1-AS1, SNORD12C, SNORD12B, SNORD12, KCNB1, PTGIS, B4GALT5, SLC9A8, SPATA2, RNF114, SNAI1, LOC100887755, UBE2V1, TMEM189-UBE2V1, TMEM189, CEBPB, LOC284751, PTPN1, MIR645, FAM65C, PARD6B, BCAS4, ADNP, DPM1, MOCS3, KCNG1, NFATC2, MIR3194, ATP9A, SALL4, ZFP64, TSHZ2, ZNF217, SUMO1P1, BCAS1, MIR4756, CYP24A1, PFDN4, DOK5, CBLN4, MC3R, FAM210B, AURKA, CSTF1, CASS4, C20orf43, GCNT7, FAM209A, FAM209B, TFAP2C, BMP7, MIR4325, SPO11, MIR5095, RAE1, MTRNR2L3, RBM38, CTCFL, PCK1, ZBP1, PMEPA1, MIR4532, C20orf85, PPP4R1L, RAB22A, VAPB, APCDD1L, LOC149773, STX16, STX16-NPEPL1, NPEPL1, MIR296, MIR298, GNAS-AS1, GNAS, TH1L, CTSZ, TUBB1, ATP5E, SLMO2-ATP5E, SLMO2, ZNF831, EDN3, PHACTR3, LOC100506384, SYCP2, FAM217B, PPP1R3D, CDH26, C20orf197, LOC284757, MIR4533, CDH4, MIR1257, TAF4, LSM14B, PSMA7, SS18L1, GTPBP5, HRH3, OSBPL2, ADRM1, LAMA5, MIR4758, RPS21, CABLES2, C20orf151, GATA5, C20orf166-AS1, C20orf166, MIR1-1, MIR133A2, SLCO4A1, LOC100127888, NTSR1, LOC100652730, C20orf20, OGFR, COL9A3, TCFL5, DPH3P1, DIDO1, C20orf11, SLC17A9, BHLHE23, LOC63930, LINC00029, LOC100144597, HAR1B, HAR1A, MIR124-3, YTHDF1, BIRC7, MIR3196, NKAIN4, FLJ16779, ARFGAP1, MIR4326, COL20A1, CHRNA4, KCNQ2, EEF1A2, PPDPF, PTK6, SRMS, C20orf195, PRIC285, GMEB2, STMN3, RTEL1, RTEL1-TNFRSF6B, TNFRSF6B, ARFRP1, ZGPAT, LIME1, SLC2A4RG, ZBTB46, ABHD16B, TPD52L2, LOC100505815, DNAJC5, MIR941-1, MIR941-3, MIR941-4, MIR941-2, UCKL1, MIR1914, MIR647, UCKL1-AS1, ZNF512B, SAMD10, PRPF6, LINC00176, SOX18, TCEA2, RGS19, OPRL1, C20orf201, NPBWR2, MYT1, PCMTD2	Ambatipudi et al., 2006; Morris et al., 2011, Pickering et al., 2013
Loss	3p26.3-p14.2	56.3	215, 174, 188, 150, 195, 207, 247, 115, 166	CHL1, CNTN6, CNTN4, CNTN4-AS2, IL5RA, TRNT1, CRBN, LRRN1, SETMAR, SUMF1, ITPR1, EGOT, LOC100507582, BHLHE40, ARL8B, EDEM1, MIR4790, GRM7, LOC100288428, LMCD1, LINC00312, C3orf32, CAV3, OXTR, RAD18, SRGAP3, THUMPD3, LOC440944, SETD5, LHFPL4, MTMR14, CPNE9, BRPF1, OGG1, CAMK1, TADA3, ARPC4, ARPC4-TTLL3, TTLL3, RPUSD3, CIDEC, JAGN1, IL17RE, IL17RC, CRELD1, PRRT3, PRRT3-AS1, EMC3, LOC401052, CIDECP, FANCD2, C3orf24, BRK1, VHL, IRAK2, TATDN2, GHRLOS2, GHRL, GHRLOS, SEC13, ATP2B2, MIR885, LINC00606, SLC6A11, SLC6A1, SLC6A1-AS1, HRH1, ATG7, VGLL4, TAMM41, SYN2, TIMP4, PPARG, TSEN2, LOC100129480, MKRN2, RAF1, TMEM40, CAND2, RPL32, SNORA7A, IQSEC1, NUP210, HDAC11, FBLN2, LOC285375, WNT7A, LOC100132526, TPRXL, CHCHD4, TMEM43, XPC, LSM3, SLC6A6, GRIP2, C3orf19, C3orf20, FGD5, FGD5-AS1, NR2C2, MRPS25, ZFYVE20, COL6A4P1, CAPN7, LOC100505696, SH3BP5, METTL6, EAF1, COLQ, MIR4270, HACL1, BTD, ANKRD28, MIR563, GALNTL2, DPH3, OXNAD1, RFTN1, DAZL, PLCL2, MIR3714, TBC1D5, LOC339862, SATB1, KCNH8, MIR4791, EFHB, RAB5A, PP2D1, KAT2B, SGOL1, SGOL1-AS1, VENTXP7, ZNF385D, UBE2E2, MIR548AC, UBE2E1, NKIRAS1, RPL15, NR1D2, LOC152024, THRB, LOC644990, MIR4792, RARB, TOP2B, MIR4442, NGLY1, OXSM, LOC285326, LRRC3B, NEK10, SLC4A7, EOMES, CMC1, AZI2, ZCWPW2, LOC645206, RBMS3, TGFBR2, GADL1, STT3B, OSBPL10, OSBPL10-AS1, ZNF860, GPD1L, CMTM8, CMTM7, CMTM6, DYNC1LI1, CNOT10, TRIM71, CCR4, GLB1, TMPPE, CRTAP, SUSD5, FBXL2, UBP1, CLASP2, PDCD6IP, ARPP21, MIR128-2, STAC, DCLK3, TRANK1, EPM2AIP1, MLH1, LRRFIP2, GOLGA4, C3orf35, ITGA9, CTDSPL, MIR26A1, VILL, PLCD1, DLEC1, ACAA1, MYD88, OXSR1, SLC22A13, SLC22A14, XYLB, LOC100128640, ACVR2B, EXOG, SCN5A, SCN10A, SCN11A, WDR48, GORASP1, TTC21A, CSRNP1, XIRP1, CX3CR1, CCR8, SLC25A38, RPSA, SNORA6, SNORA62, MOBP, MYRIP, FLJ33065, EIF1B, ENTPD3, ENTPD3-AS1, RPL14, ZNF619, ZNF620, ZNF621, CTNNB1, ULK4, TRAK1, CCK, LYZL4, VIPR1, SEC22C, SS18L2, NKTR, ZBTB47, KBTBD5, HHATL, CCDC13, CCDC13-AS1, HIGD1A, CCBP2, CYP8B1, ZNF662, KRBOX1, FAM198A, GTDC2, SNRK, SNRK-AS1, ANO10, ABHD5, MIR138-1, C3orf77, C3orf23, ZNF445, ZNF167, ZNF660, ZNF197, ZNF35, ZNF502, ZNF501, KIAA1143, KIF15, MIR564, TMEM42, TGM4, ZDHHC3, EXOSC7, CLEC3B, CDCP1, TMEM158, LARS2, LARS2-AS1, LIMD1, LIMD1-AS1, SACM1L, SLC6A20, LZTFL1, CCR9, FYCO1, CXCR6, XCR1, CCR1, CCR3, CCR2, CCR5, CCRL2, LTF, RTP3, LRRC2, TDGF1, LOC100132146, ALS2CL, TMIE, PRSS50, PRSS46, PRSS45, PRSS42, MYL3, PTH1R, CCDC12, NBEAL2, NRADDP, SETD2, FLJ39534, KIF9, KLHL18, PTPN23, SCAP, C3orf75, CSPG5, SMARCC1, DHX30, MIR1226, MAP4, CDC25A, MIR4443, CAMP, ZNF589, NME6, SPINK8, FBXW12, PLXNB1, CCDC51, TMA7, ATRIP, TREX1, SHISA5, PFKFB4, UCN2, COL7A1, MIR711, UQCRC1, TMEM89, SLC26A6, CELSR3, MIR4793, NCKIPSD, IP6K2, PRKAR2A, SLC25A20, C3orf71, ARIH2, P4HTM, WDR6, DALRD3, MIR425, NDUFAF3, MIR191, IMPDH2, QRICH1, QARS, USP19, LAMB2, LAMB2P1, CCDC71, KLHDC8B, LOC646498, CCDC36, C3orf62, MIR4271, USP4, GPX1, RHOA, TCTA, AMT, NICN1, DAG1, BSN-AS2, BSN, APEH, MST1, RNF123, AMIGO3, GMPPB, IP6K1, CDHR4, FAM212A, UBA7, MIR5193, TRAIP, CAMKV, MST1R, MON1A, RBM6, RBM5, LUST, SEMA3F, GNAT1, SLC38A3, GNAI2, SEMA3B, C3orf45, IFRD2, HYAL3, NAT6, HYAL1, HYAL2, TUSC2, RASSF1, ZMYND10, NPRL2, CYB561D2, TMEM115, CACNA2D2, C3orf18, HEMK1, CISH, MAPKAPK3, MIR4787, DOCK3, MANF, RBM15B, VPRBP, RAD54L2, TEX264, GRM2, IQCF6, IQCF4, IQCF3, IQCF2, IQCF5, IQCF1, RRP9, PARP3, GPR62, PCBP4, ABHD14B, ABHD14A, ABHD14A-ACY1, ACY1, RPL29, DUSP7, C3orf74, POC1A, ALAS1, TLR9, TWF2, PPM1M, WDR82, MIRLET7G, GLYCTK, MIR135A1, DNAH1, BAP1, PHF7, SEMA3G, TNNC1, NISCH, STAB1, NT5DC2, C3orf78, PBRM1, GNL3, SNORD19, SNORD19B, SNORD69, GLT8D1, SPCS1, NEK4, ITIH1, ITIH3, ITIH4, MUSTN1, TMEM110-MUSTN1, TMEM110, SFMBT1, RFT1, PRKCD, TKT, DCP1A, CACNA1D, CHDH, IL17RB, ACTR8, SELK, CACNA2D3, ESRG, CACNA2D3-AS1, LRTM1, WNT5A, ERC2, ERC2-IT1, MIR3938, CCDC66, FAM208A, ARHGEF3, ARHGEF3-AS1, SPATA12, IL17RD, HESX1, APPL1, ASB14, DNAH12, PDE12, ARF4, FAM116A, SLMAP, FLNB, DNASE1L3, ABHD6, RPP14, PXK, PDHB, KCTD6, ACOX2, FAM107A, FAM3D, C3orf67, FHIT, PTPRG	Ambatipudi et al., 2006; Morris et al., 2011, Pickering et al., 2013
Loss	3p14.2-p12.3	56.3	215, 174, 188, 150, 195, 207, 247, 204, 166	LOC100506994, C3orf14, FEZF2, CADPS, LOC285401, SYNPR, SYNPR-AS1, SNTN, C3orf49, THOC7, ATXN7, PSMD6-AS2, PSMD6, PRICKLE2-AS1, LOC100287879, PRICKLE2, PRICKLE2-AS2, PRICKLE2-AS3, ADAMTS9, ADAMTS9-AS2, MIR548A2, MAGI1, MAGI1-AS1, SLC25A26, LRIG1, KBTBD8, MIR4272, SUCLG2, FAM19A1, FAM19A4, EOGT, TMF1, MIR3136, UBA3, ARL6IP5, LMOD3, FRMD4B, MITF, FOXP1, MIR1284, EIF4E3, GPR27, PROK2, LOC201617, RYBP, SHQ1, GXYLT2, PPP4R2, EBLN2, PDZRN3, CNTN3, MIR4444-1, FAM86DP, MIR1324, FLJ20518, FRG2C, LOC401074, ZNF717, MIR4273, ROBO2, ROBO1	Ambatipudi et al., 2006; Morris et al., 2011, Pickering et al., 2013
Loss	3p12.3-p11.1	50.0	215, 188, 150, 195, 207, 247, 204, 166	GBE1, LOC440970, CADM2, MIR5688, CADM2-AS2, RNU6-69, VGLL3, MIR4795, CHMP2B, POU1F1, HTR1F, CGGBP1, ZNF654, C3orf38, EPHA3	Ambatipudi et al., 2006; Morris et al., 2011, Pickering et al., 2013
Loss	4q32.1-q32.3	43.8	215, 174, 150, 196, 207, 136, 204,	RXFP1, C4orf46, ETFDH, PPID, FNIP2, C4orf45, MIR3688-1, MIR3688-2, RAPGEF2, FSTL5, NAF1, NPY1R, NPY5R, TKTL2, TMA16, Mar-01, ANP32C, MIR5684, LOC100505989, LOC100506013, TRIM61, FAM218A, TRIM60	
Loss	4q32.3-q35.2	50.0	215, 174, 150, 196, 214, 207, 136, 204	TMEM192, KLHL2, GK3P, MSMO1, CPE, MIR578, TLL1, SPOCK3, ANXA10, DDX60, DDX60L, PALLD, CBR4, SH3RF1, NEK1, CLCN3, C4orf27, LOC100506085, MFAP3L, AADAT, HSP90AA6P, LOC100506122, GALNTL6, GALNT7, HMGB2, SAP30, SCRG1, HAND2, NBLA00301, FBXO8, CEP44, MIR4276, HPGD, GLRA3, ADAM29, GPM6A, WDR17, SPATA4, ASB5, SPCS3, VEGFC, NEIL3, AGA, LOC285501, LINC00290, MGC45800, MIR1305, ODZ3, DCTD, FAM92A1P2, WWC2-AS2, WWC2, CLDN22, CLDN24, CDKN2AIP, LOC389247, ING2, RWDD4, TRAPPC11, STOX2, ENPP6, LOC728175, IRF2, CASP3, CCDC111, MLF1IP, ACSL1, SLED1, LOC731424, MIR3945, LOC100506229, HELT, SLC25A4, KIAA1430, SNX25, LRP2BP, ANKRD37, UFSP2, C4orf47, CCDC110, PDLIM3, SORBS2, TLR3, FAM149A, FLJ38576, CYP4V2, KLKB1, F11, LOC285441, MTNR1A, FAT1, LOC339975, ZFP42, TRIML2, TRIML1, LOC401164, HSP90AA4P, FRG1, FRG2, LOC100288255	Ambatipudi et al., 2006; Morris et al., 2011; Pickering et al., 2013
Loss	8p23.3-p11.1	81.3	215, 153, 174, 188, 150, 195, 156, 196, 214, 207, 136, 204, 166	RPL23AP53, ZNF596, FBXO25, C8orf42, ERICH1, LOC286083, DLGAP2, CLN8, MIR596, ARHGEF10, KBTBD11, MYOM2, CSMD1, LOC100287015, MCPH1, ANGPT2, AGPAT5, MIR4659A, MIR4659B, XKR5, LOC100652791, DEFB1, DEFA6, DEFA4, DEFA10P, DEFA1, DEFA1B, DEFT1P2, DEFT1P, DEFA3, DEFA5, LOC349196, FAM66B, DEFB109P1B, USP17L1P, USP17L4, ZNF705G, DEFB4B, DEFB103B, DEFB103A, SPAG11B, DEFB104A, DEFB104B, DEFB106B, DEFB106A, DEFB105A, DEFB105B, DEFB107B, DEFB107A, FAM90A7P, FAM90A10P, SPAG11A, DEFB4A, LOC100132396, FAM66E, USP17L8, USP17L3, MIR548I3, FLJ10661, SGK223, CLDN23, MFHAS1, ERI1, MIR4660, PPP1R3B, LOC157273, TNKS, MIR597, LINC00599, MIR124-1, MSRA, PRSS55, RP1L1, C8orf74, SOX7, PINX1, MIR1322, XKR6, MIR598, MTMR9, SLC35G5, TDH, C8orf12, FAM167A, BLK, LINC00208, GATA4, NEIL2, FDFT1, CTSB, DEFB136, DEFB135, DEFB134, DEFB130, LOC100133267, ZNF705D, LOC392196, USP17L7, USP17L2, FAM90A2P, FAM86B1, FAM66A, LOC649352, DEFB109P1, FAM86B2, LOC100506990, LOC729732, LONRF1, MIR3926-1, MIR3926-2, LOC340357, KIAA1456, DLC1, C8orf48, SGCZ, MIR383, TUSC3, MSR1, FGF20, EFHA2, ZDHHC2, CNOT7, VPS37A, MTMR7, SLC7A2, PDGFRL, MTUS1, FGL1, PCM1, ASAH1, NAT1, NAT2, PSD3, LOC100128993, SH2D4A, CSGALNACT1, INTS10, LPL, SLC18A1, ATP6V1B2, LZTS1, LZTS1-AS1, LOC286114, GFRA2, DOK2, XPO7, NPM2, FGF17, EPB49, FAM160B2, NUDT18, HR, REEP4, LGI3, SFTPC, BMP1, PHYHIP, MIR320A, POLR3D, PIWIL2, SLC39A14, PPP3CC, SORBS3, PDLIM2, C8orf58, KIAA1967, BIN3, FLJ14107, EGR3, PEBP4, RHOBTB2, TNFRSF10B, LOC286059, LOC254896, TNFRSF10C, TNFRSF10D, TNFRSF10A, LOC389641, CHMP7, R3HCC1, LOXL2, LOC100507156, ENTPD4, SLC25A37, NKX3-1, NKX2-6, STC1, ADAM28, ADAMDEC1, ADAM7, NEFM, NEFL, DOCK5, GNRH1, KCTD9, CDCA2, EBF2, PPP2R2A, BNIP3L, PNMA2, DPYSL2, ADRA1A, STMN4, TRIM35, PTK2B, CHRNA2, EPHX2, CLU, SCARA3, MIR3622B, MIR3622A, CCDC25, ESCO2, PBK, SCARA5, MIR4287, NUGGC, ELP3, PNOC, ZNF395, FBXO16, FZD3, MIR4288, EXTL3, INTS9, HMBOX1, KIF13B, DUSP4, LINC00589, LOC286135, MIR3148, MIR548O2, TMEM66, LEPROTL1, MBOAT4, DCTN6, LOC100128750, RBPMS, GTF2E2, LOC100507341, GSR, UBXN8, PPP2CB, TEX15, PURG, WRN, NRG1, NRG1-IT3, FUT10, MAK16, TTI2, RNF122, DUSP26, UNC5D, KCNU1, ZNF703, ERLIN2, LOC728024, PROSC, GPR124, BRF2, RAB11FIP1, GOT1L1, ADRB3, EIF4EBP1, ASH2L, STAR, LSM1, BAG4, DDHD2, PPAPDC1B, WHSC1L1, LETM2, FGFR1, C8orf86, RNF5P1, TACC1, PLEKHA2, HTRA4, TM2D2, ADAM9, ADAM32, ADAM5P, ADAM3A, LOC100130964, ADAM18, ADAM2, IDO1, IDO2, C8orf4, ZMAT4, SFRP1, GOLGA7, GINS4, AGPAT6, NKX6-3, ANK1, MIR486, KAT6A, AP3M2, PLAT, IKBKB, POLB, DKK4, VDAC3, SLC20A2, C8orf40, CHRNB3, CHRNA6, THAP1, RNF170, MIR4469, HOOK3, FNTA, SGK196, HGSNAT, POTEA	Ambatipudi et al., 2006; Morris et al., 2011, Pickering et al., 2013
Loss	10p15.3-p11.1	43.8	215, 153, 174, 150, 156, 207, 136	TUBB8, ZMYND11, DIP2C, MIR5699, C10orf108, LARP4B, GTPBP4, IDI2, IDI2-AS1, IDI1, WDR37, LINC00200, ADARB2, ADARB2-AS1, LOC282980, LOC399708, PFKP, PITRM1, LOC100507034, KLF6, LOC100216001, LOC338588, AKR1E2, TAKR, AKR1C1, AKR1C2, AKR1C3, AKR1CL1, AKR1C4, UCN3, TUBAL3, NET1, CALML5, CALML3, ASB13, FAM208B, GDI2, ANKRD16, FBXO18, IL15RA, IL2RA, RBM17, PFKFB3, MIR3155A, MIR3155B, LOC399715, PRKCQ, LOC439949, LOC100507127, SFMBT2, ITIH5, ITIH2, KIN, ATP5C1, TAF3, FLJ45983, GATA3, SFTA1P, LOC254312, CELF2, LOC439950, USP6NL, ECHDC3, C10orf47, LOC219731, UPF2, DHTKD1, SEC61A2, NUDT5, CDC123, CAMK1D, MIR4480, LOC283070, CCDC3, OPTN, MCM10, UCMA, PHYH, SEPHS1, BEND7, PRPF18, FRMD4A, MIR1265, FAM107B, CDNF, HSPA14, SUV39H2, DCLRE1C, MEIG1, OLAH, ACBD7, C10orf111, RPP38, NMT2, LOC100192204, FAM171A1, ITGA8, FAM188A, PTER, C1QL3, RSU1, CUBN, TRDMT1, VIM, ST8SIA6, LOC100128098, PTPLA, STAM, TMEM236, MRC1, MIR511-2, MIR511-1, SLC39A12, LOC100129213, CACNB2, NSUN6, ARL5B, PLXDC2, MIR4675, NEBL, C10orf113, LOC100128511, C10orf114, MIR1915, SKIDA1, MLLT10, DNAJC1, EBLN1, LOC100130992, COMMD3, COMMD3-BMI1, BMI1, SPAG6, LOC100499489, PIP4K2A, ARMC3, MSRB2, PTF1A, C10orf67, OTUD1, KIAA1217, PRINS, MIR603, ARHGAP21, PRTFDC1, ENKUR, THNSL1, GPR158-AS1, GPR158, MYO3A, GAD2, APBB1IP, LINC00264, LOC731789, PDSS1, ABI1, LINC00202, ANKRD26, YME1L1, MASTL, ACBD5, LRRC37A6P, PTCHD3, RAB18, MKX, ARMC4, MPP7, LOC220906, WAC, MIR5586, BAMBI, LOC100507605, LYZL1, LOC387647, SVIL, MIR604, MIR938, KIAA1462, MTPAP, LOC729668, MAP3K8, LYZL2, ZNF438, ZEB1-AS1, ZEB1, ARHGAP12, KIF5B, EPC1, CCDC7, C10orf68, ITGB1, NRP1, LOC100505583, PARD3, CUL2, CREM, CCNY, GJD4, FZD8, MIR4683, ANKRD30A, MTRNR2L7, ZNF248, LOC100419868, ZNF25, ZNF33A, ZNF37A, LOC100129055	Ambatipudi et al., 2006
Loss	11q22.3-q23.3	31.3	188, 195, 247, 204, 166	DYNC2H1, MIR4693, PDGFD, DDI1, CASP12, LOC643733, CASP4, CASP5, CASP1, CARD16, CARD17, CARD18, GRIA4, MSANTD4, KBTBD3, AASDHPPT, GUCY1A2, CWF19L2, ALKBH8, ELMOD1, LOC643923, SLN, SLC35F2, RAB39A, CUL5, ACAT1, NPAT, ATM, C11orf65, KDELC2, EXPH5, DDX10, C11orf87, ZC3H12C, RDX, FDX1, ARHGAP20, C11orf53, C11orf92, C11orf93, MIR4491, POU2AF1, LOC100132078, BTG4, MIR34B, MIR34C, C11orf88, LAYN, SIK2, PPP2R1B, ALG9, FDXACB1, C11orf1, CRYAB, HSPB2, HSPB2-C11orf52, C11orf52, DIXDC1, DLAT, PIH1D2, C11orf57, TIMM8B, SDHD, IL18, TEX12, BCO2, PTS, C11orf34, NCAM1, LOC100288346, TTC12, ANKK1, DRD2, MIR4301, TMPRSS5, ZW10, CLDN25, USP28, HTR3B, HTR3A, ZBTB16, NNMT, C11orf71, RBM7, REXO2, NXPE1, NXPE4, NXPE2, CADM1, LOC283143, BUD13, ZNF259, APOA5, APOA4, APOC3, APOA1, SIK3, SIK3-IT1, PAFAH1B2, SIDT2, LOC100652768, TAGLN, PCSK7, RNF214, BACE1, BACE1-AS, CEP164, DSCAML1, FXYD2, FXYD6-FXYD2, FXYD6, TMPRSS13, IL10RA, LOC100526771, TMPRSS4, SCN4B, SCN2B, AMICA1, MPZL3, MPZL2, CD3E, CD3D, CD3G, UBE4A, LOC100131626, ATP5L, MLL, TTC36, TMEM25, IFT46, ARCN1, PHLDB1, TREH, DDX6, CXCR5, BCL9L, MIR4492, UPK2, FOXR1, CCDC84, RPL23AP64, RPS25, TRAPPC4, MIR3656, SLC37A4, HYOU1, VPS11, HMBS, H2AFX, DPAGT1, C2CD2L, HINFP, ABCG4, NLRX1, PDZD3, CCDC153, CBL, MCAM, RNF26, MFRP, C1QTNF5, USP2, LOC100499227, THY1, PVRL1, TRIM29, OAF, POU2F3, LOC649133, TMEM136	Ambatipudi et al., 2006, Pickering et al., 2013
Loss	18q11.2-q12.3	50.0	215, 150, 195, 156, 196, 115, 204, 166	SS18, PSMA8, TAF4B, KCTD1, LOC728606, AQP4, CHST9-AS1, CHST9, CDH2, MIR302F, DSC3, DSC2, DSC1, DSG1, DSG4, DSG3, DSG2, LOC100652770, TTR, B4GALT6, SLC25A52, TRAPPC8, RNF125, RNF138, MEP1B, FAM59A, WBP11P1, KLHL14, C18orf34, ASXL3, NOL4, DTNA, MAPRE2, ZNF397, ZSCAN30, ZNF271, ZNF24, ZNF396, INO80C, MIR3975, GALNT1, MIR187, C18orf21, RPRD1A, SLC39A6, ELP2, MOCOS, FHOD3, TPGS2, KIAA1328, CELF4, MIR4318, LOC647946, MIR5583-2, MIR5583-1	Ambatipudi et al., 2006
Loss	18q12.3-q23	56.3	215, 153, 150, 195, 156, 196, 115, 204, 166	KC6, PIK3C3, LOC284260, RIT2, SYT4, SETBP1, MIR4319, SLC14A2, SLC14A1, SIGLEC15, EPG5, PSTPIP2, ATP5A1, HAUS1, C18orf25, RNF165, LOXHD1, ST8SIA5, PIAS2, KATNAL2, TCEB3CL, LOC100506888, TCEB3C, TCEB3B, HDHD2, IER3IP1, SMAD2, ZBTB7C, CTIF, MIR4743, SMAD7, DYM, MIR4744, C18orf32, RPL17-C18ORF32, MIR1539, RPL17, SNORD58C, SNORD58A, SNORD58B, LIPG, ACAA2, SCARNA17, MYO5B, MIR4320, CCDC11, MBD1, CXXC1, SKA1, MAPK4, MRO, ME2, ELAC1, SMAD4, MEX3C, LOC100287225, DCC, MBD2, SNORA37, POLI, STARD6, C18orf54, C18orf26, RAB27B, CCDC68, TCF4, MIR4529, LOC100505474, TXNL1, WDR7, LINC-ROR, BOD1L2, ST8SIA3, ONECUT2, FECH, NARS, LOC100505549, ATP8B1, NEDD4L, MIR122, MIR3591, ALPK2, MALT1, ZNF532, LOC390858, SEC11C, GRP, RAX, CPLX4, LMAN1, CCBE1, PMAIP1, MC4R, CDH20, RNF152, PIGN, KIAA1468, TNFRSF11A, ZCCHC2, PHLPP1, BCL2, KDSR, VPS4B, SERPINB5, SERPINB12, SERPINB13, SERPINB4, SERPINB3, SERPINB11, SERPINB7, SERPINB2, SERPINB10, HMSD, SERPINB8, LINC00305, LOC284294, LOC400654, CDH7, CDH19, MIR5011, DSEL, LOC643542, TMX3, CCDC102B, DOK6, CD226, RTTN, SOCS6, LOC100505776, CBLN2, NETO1, LOC400655, LOC100505817, FBXO15, TIMM21, CYB5A, C18orf63, FAM69C, CNDP2, CNDP1, LOC400657, ZNF407, ZADH2, TSHZ1, C18orf62, LOC339298, ZNF516, LOC284276, LOC100131655, ZNF236, MBP, GALR1, SALL3, ATP9B, NFATC1, CTDP1, KCNG2, PQLC1, HSBP1L1, TXNL4A, RBFA, ADNP2, PARD6G-AS1, PARD6G	Ambatipudi et al., 2006; Morris et al., 2011, Pickering et al., 2013
Loss	22q12.1-q13.33	31.3	215, 188, 150, 115, 166	SEZ6L, ASPHD2, HPS4, SRRD, TFIP11, TPST2, MIR548J, CRYBB1, CRYBA4, MIAT, MN1, PITPNB, TTC28-AS1, MIR3199-1, MIR3199-2, TTC28, CHEK2, HSCB, CCDC117, XBP1, ZNRF3, ZNRF3-AS1, C22orf31, KREMEN1, EMID1, RHBDD3, EWSR1, GAS2L1, RASL10A, AP1B1, MIR3653, SNORD125, RFPL1-AS1, RFPL1, NEFH, THOC5, NIPSNAP1, NF2, CABP7, ZMAT5, UQCR10, ASCC2, MTMR3, HORMAD2, LIF, OSM, GATSL3, TBC1D10A, SF3A1, CCDC157, KIAA1656, RNF215, SEC14L2, MTFP1, SEC14L3, SDC4P, SEC14L4, SEC14L6, GAL3ST1, PES1, TCN2, SLC35E4, DUSP18, OSBP2, MIR3200, MORC2-AS1, MORC2, TUG1, SMTN, SELM, INPP5J, PLA2G3, MIR3928, RNF185, LIMK2, PIK3IP1, PATZ1, DRG1, EIF4ENIF1, SFI1, PISD, PRR14L, DEPDC5, C22orf24, YWHAH, SLC5A1, AP1B1P1, C22orf42, RFPL2, SLC5A4, RFPL3, RFPL3-AS1, LOC339666, C22orf28, BPIFC, FBXO7, SYN3, TIMP3, LARGE, MIR4764, LOC100506195, ISX, HMGXB4, TOM1, MIR3909, HMOX1, MCM5, RASD2, MB, APOL6, APOL5, RBFOX2, APOL3, APOL4, APOL2, APOL1, MYH9, TXN2, FOXRED2, EIF3D, CACNG2, IFT27, PVALB, NCF4, CSF2RB, LOC100506241, TEX33, TST, MPST, KCTD17, TMPRSS6, IL2RB, C1QTNF6, SSTR3, RAC2, CYTH4, ELFN2, MFNG, CARD10, CDC42EP1, LGALS2, GGA1, SH3BP1, PDXP, LGALS1, NOL12, TRIOBP, H1F0, GCAT, GALR3, ANKRD54, MIR658, MIR659, EIF3L, MICALL1, C22orf23, POLR2F, SOX10, MIR4534, PICK1, SLC16A8, BAIAP2L2, PLA2G6, MAFF, TMEM184B, CSNK1E, LOC400927, KCNJ4, KDELR3, DDX17, DMC1, LOC646851, CBY1, TOMM22, JOSD1, GTPBP1, SUN2, DNAL4, NPTXR, CBX6, APOBEC3A, APOBEC3A_B, APOBEC3B, APOBEC3C, APOBEC3D, APOBEC3F, APOBEC3G, APOBEC3H, CBX7, PDGFB, RPL3, SNORD83B, SNORD83A, RNU86, SNORD43, SYNGR1, TAB1, LOC100506472, MGAT3, SMCR7L, ATF4, RPS19BP1, CACNA1I, ENTHD1, GRAP2, FAM83F, LOC100130899, TNRC6B, ADSL, SGSM3, MKL1, MCHR1, SLC25A17, MIR4766, ST13, XPNPEP3, DNAJB7, RBX1, MIR1281, EP300, L3MBTL2, CHADL, RANGAP1, ZC3H7B, TEF, TOB2, PHF5A, ACO2, POLR3H, CSDC2, PMM1, DESI1, XRCC6, NHP2L1, C22orf46, MEI1, CCDC134, SREBF2, MIR33A, SHISA8, TNFRSF13C, CENPM, BK250D10.8, Sep-03, WBP2NL, NAGA, FAM109B, C22orf32, NDUFA6, LOC100132273, CYP2D6, CYP2D7P1, TCF20, LOC388906, NFAM1, SERHL, RRP7A, SERHL2, RRP7B, POLDIP3, RNU12, CYB5R3, ATP5L2, A4GALT, ARFGAP3, PACSIN2, TTLL1, BIK, MCAT, TSPO, TTLL12, SCUBE1, MPPED1, EFCAB6-AS1, EFCAB6, SULT4A1, PNPLA5, PNPLA3, SAMM50, PARVB, PARVG, KIAA1644, LDOC1L, LINC00207, LINC00229, PRR5, PRR5-ARHGAP8, ARHGAP8, PHF21B, LOC100506714, NUP50, KIAA0930, MIR1249, UPK3A, FAM118A, SMC1B, RIBC2, FBLN1, ATXN10, MIR4762, WNT7B, LOC730668, LOC100271722, C22orf26, LOC150381, MIRLET7BHG, MIR3619, MIRLET7A3, MIR4763, MIRLET7B, PPARA, C22orf40, PKDREJ, TTC38, CN5H6.4, GTSE1, TRMU, CELSR1, GRAMD4, CERK, TBC1D22A, LOC339685, FLJ46257, MIR3201, FAM19A5, LOC284933, MIR4535, LOC100128946, C22orf34, BRD1, LOC90834, ZBED4, ALG12, CRELD2, PIM3, IL17REL, MLC1, MOV10L1, PANX2, TRABD, SELO, TUBGCP6, HDAC10, MAPK12, MAPK11, PLXNB2, FAM116B, PPP6R2, SBF1, ADM2, MIOX, LMF2, NCAPH2, SCO2, TYMP	
